# Suppression of large t antigen-stimulated CD4^+^CD25^+^ and CD8^+^IFN-γ^+^ T cells is strongly associated with low level BK viremia in kidney transplant recipients

**DOI:** 10.3389/fmed.2025.1662833

**Published:** 2025-11-14

**Authors:** Wilasinee Saisorn, Thunyatorn Wuttiputhanun, Jakapat Vanichanan, Kamonwan Jutivorakool, Natavudh Townamchai, Yingyos Avihingsanon, Asada Leelahavanichkul, Suwasin Udomkarnjananun

**Affiliations:** 1 Department of Microbiology, Faculty of Medicine, Center of Excellence on Translational Research in Inflammation and Immunology, Chulalongkorn University, Bangkok, Thailand; 2 Division of Nephrology, Department of Medicine, Faculty of Medicine, Chulalongkorn University and King Chulalongkorn Memorial Hospital, The Thai Red Cross Society, Bangkok, Thailand; 3 Excellence Center for Organ Transplantation, King Chulalongkorn Memorial Hospital, The Thai Red Cross Society, Bangkok, Thailand; 4 Faculty of Medicine, Renal Immunology and Renal Transplant Center of Excellence, Chulalongkorn University, Bangkok, Thailand; 5 Division of Infectious Disease, Department of Medicine, Faculty of Medicine, Chulalongkorn University and King Chulalongkorn Memorial Hospital, The Thai Red Cross Society, Bangkok, Thailand; 6 Immunology Unit, Department of Microbiology, Chulalongkorn University, Bangkok, Thailand

**Keywords:** activated T cells, BK polyomavirus, large T antigen, kidney transplantation, infection-immunology

## Abstract

**Background:**

BK polyomavirus (BKPyV) infection following kidney transplantation results from over-suppression of cellular immunity. Currently, there is no established, clinically applicable immunological assay that comprehensively monitors cellular immune responses against BKPyV, incorporating both cytokine production and T cell activation markers. Our study aimed to comprehensively assess both cytokine production and surface activation markers to differentiate kidney transplant recipients (KTR) with low-level (<3,000 copies/mL) BKPyV viremia from those without viremia.

**Methods:**

Thirty-six participants were enrolled, comprising KTR with (BK) and without BKPyV viremia (nBK), alongside healthy controls (HC). Peripheral blood mononuclear cells (PBMC) were stimulated using BKPyV viral capsid protein-1 (VP1) or large-T-antigen (LTA), with and without CD28/CD49d co-stimulatory antibodies. Outcomes included expression of IL-2, IFN-γ, TNF-α, CD25, CD134, CD137, and CD154. Candidate markers were evaluated by calculating the area under the receiver operating characteristic curve (AUROC) for diagnosing BKPyV viremia.

**Results:**

VP1- or LTA-stimulated CD4^+^ and CD8^+^ T cells showed optimal discriminatory power between BK and nBK groups when co-stimulated with CD28/CD49d. VP1-stimulated CD4^+^ cells differed significantly between groups in IL-2, TNF-α, CD25, and CD137, while CD8^+^ cells differed significantly in IFN-γ and CD25. LTA-stimulated CD4^+^ cells showed significant differences in TNF-α and CD25, and CD8^+^ cells differed significantly in IFN-γ and CD25. LTA-stimulated CD4^+^CD25^+^ and CD8^+^IFN-γ^+^ cells provided significant AUROC values (0.823, 95%CI 0.657–0.989, *p* = 0.030; and 0.833, 95%CI 0.678–0.989, *p* = 0.028, respectively) at a cutoff of > 0.2% positive cells.

**Conclusion:**

LTA-stimulated CD4^+^CD25^+^ and CD8^+^IFN-γ^+^ T cells differentiated KTR with and without low-level BKPyV viremia, representing promising markers for early clinical diagnostics and future studies.

## Highlights

- Although several studies have demonstrated an association between BK polyomavirus (BKPyV)-specific cellular immune responses and BKPyV infection in kidney transplant recipients (KTR), no immunological assays are currently established for routine clinical use.
- This study comprehensively assessed BKPyV-specific cellular immune responses by stimulating cells with viral capsid protein-1 (VP1) and large T antigen (LTA), both with and without CD28/CD49d co-stimulatory antibodies, and evaluated cytokines (IL-2, IFN-γ, TNF-α) as well as surface markers of activated T cells (CD25, CD134, CD137, and CD154) for their association with BKPyV viremia in KTR. Only KTR with low-level (< 3,000 copies/mL) BKPyV viremia were included to focus on the early clinical course of BKPyV infection and to identify potential early immune markers.
- KTR with BKPyV viremia (BK group) exhibited significantly lower percentages of positive cells for multiple markers compared to healthy non-transplant controls (HC). Notably, only LTA, in combination with CD28/CD49d co-stimulation, demonstrated sufficient discriminatory power to differentiate between BK and non-viremic (nBK) KTR groups in both CD4^+^ and CD8^+^ T cells.
- A cutoff value of > 0.2% positive cells (after background subtraction with a negative unstimulated control) for LTA-stimulated CD4^+^CD25^+^ and CD8^+^IFN-γ^+^ T cells demonstrated potential for application in future clinical studies and could serve as a cost-effective diagnostic tool.

## Introduction

BK polyomavirus (BKPyV) is a significant complication following kidney transplantation. Approximately 30% of kidney transplant recipients (KTR) develop BKPyV viruria, 10–15% develop BKPyV viremia, and 3–5% eventually progress to BK polyomavirus-associated nephropathy (BKPyVAN), which markedly reduces kidney allograft survival (1–4). KTR with BKPyVAN experience an allograft loss rate of 10–50%, depending on the pathological severity (5, 6). Current evidence and recommendations indicate that the only effective treatment for BKPyVAN is the reduction of immunosuppression (7, 8). However, the success of this strategy in achieving viral clearance varies widely across studies, ranging from 20 to 80% (9). Consequently, preventing the development of BKPyV viremia or BKPyVAN is the optimal goal in managing BKPyV infection.

Several risk factors for BKPyV viremia and BKPyVAN have been established, including mismatches between donor and recipient BKPyV serostatus and genotypes, older age and male gender in recipients, low recipient neutralizing antibody levels, and certain human leukocyte antigen (HLA) types (7). Importantly, the intensity of immunosuppressive therapy significantly impacts cellular immunity against BKPyV (10–12). BKPyV-specific cellular immunity is crucial for controlling viral replication and promoting viral clearance via T-cell responses (12). Previous studies have shown that KTR with active BKPyV infection exhibit lower BKPyV-specific T-cell responses compared to those who never develop BKPyV infection or who have cleared the virus (10, 13–17). However, these studies have primarily utilized flow cytometry or enzyme-linked immunospot (ELISPOT) assays that focus solely on cytokine responses, without assessing other aspects such as the markers of activated T-cells (18–23). This limitation hampers a comprehensive understanding of BKPyV-specific cellular immunity. Additionally, the protocols used in each study varied. Some studies utilized only the BKPyV antigen (viral capsid protein 1 [VP1] and/or large T antigen [LTA]), while others added costimulatory antibodies (CD28 and/or CD49d) to enhance the cellular immune response (14–17, 24–27). This variability limits the interpretation and clinical implications of these tests.

This study aimed to compare BKPyV-specific T-cell responses using traditional cytokine analyses—including interleukin-2 (IL-2), interferon-γ (IFN-γ), and tumor necrosis factor-α (TNF-α)—with markers of activated T-cells, including CD25, CD134, CD137, and CD154 (18–23). These comparisons were conducted among three groups: KTR with active low-level BKPyV infection (defined as BKPyV viremia < 3,000 copies/mL at the time of first diagnosis and sample collection), KTR without BKPyV infection, and non-transplant healthy controls. The objective was to identify the most effective combination of immune markers for distinguishing KTRs with BKPyV infection, thereby enhancing post-transplant surveillance strategies in the early clinical course of infection.

## Materials and methods

### Study population and overview

This study was conducted at King Chulalongkorn Memorial Hospital, a tertiary transplant center in Bangkok, Thailand. KTR aged 18 years or older were included. The screening protocol for BKPyV involved monthly plasma tests until 9 months post-transplantation, followed by testing every 3 months until 2 years, in accordance with international guidelines on BKPyV management (7). KTR who developed allograft dysfunction were also evaluated for BKPyV viremia. At our center, the management of BKPyV viremia consists of reducing the mycophenolic acid (MPA) dosage by 50% and lowering the tacrolimus pre-dose concentration (C_0_) to 4–6 ng/mL when plasma BKPyV levels reach 10,000 copies/mL or 1,000 copies/mL on at least two occasions, 2 weeks apart. If, after 4 weeks of these interventions, BKPyV levels do not decrease by 0.5 log_10_ copies/mL, MPA is switched to everolimus with a target C_0_ of 4–8 ng/mL, and tacrolimus C_0_ is further reduced to 2–4 ng/mL. The prednisolone dosage is maintained at a maximum of 5 mg/day during detectable BKPyV viremia.

In KTR with BKPyV viremia, whole blood was drawn into heparinized tubes upon detection of plasma BKPyV, and the sample collected on the day of this first viremia diagnosis was used for cell isolation. All participants provided informed consent for blood collection. Simultaneously, sex-, age-, and time after transplant-matched KTR without BKPyV viremia were enrolled in the non-BKPyV viremia group. Since the study focused on identifying potential immunological markers for the early detection of BKPyV infection or reactivation, only KTR with low-level BKPyV viremia (<3,000 copies/mL) at the time of initial diagnosis were included in the BKPyV group. All samples were collected at this initial time point. The definition of presumptive BKPyVAN is BKPyV viremia > 10,000 copies/mL (7). Our study aimed to detect infection earlier, before nephropathy develops. Accordingly, we defined low-level BKPyV viremia as < 3,000 copies/mL, based on the premise that earlier identification of altered immune regulation would provide greater clinical benefit for KTRs. This cutoff was chosen to avoid being too late (i.e., > 10,000 copies/mL) and not so early (i.e., < 1,000 copies/mL) that its significance remains controversial.

Only KTR receiving maintenance immunosuppression with tacrolimus (immediate-release Prograf^®^, target C_0_ 5–10 ng/mL), MPA [equivalent to mycophenolate mofetil (MMF) at 1,000–2,000 mg/day], and prednisolone were eligible for inclusion in both the BKPyV viremia (BK) and non-BKPyV viremia (nBK) groups. Only uncomplicated BKPyV viremia cases (no desensitization protocol, no rejection, no history of other infections) were included. For the nBK group, we selected clinically stable KTRs without any post-transplant complications (i.e., good postoperative graft function and stable follow-up). Additionally, healthy controls (HC) with no medical comorbidities and not taking any medications were included as a biological reference group. Whole blood samples from the nBK and HC groups were collected and processed for cell isolation.

### PBMCs isolation

Peripheral blood mononuclear cells (PBMCs) were isolated from whole blood collected in heparinized tubes. The blood was layered onto Lymphoprep™ (STEMCELL Technologies, Serumwerk Bernburg AG, Germany) in a 1:1 ratio and centrifuged at 1,900 rpm for 30 min at room temperature without applying deceleration force. The PBMC layer was then collected and washed with RPMI 1,640 medium (Thermo Scientific, MA, United States) supplemented with 10% human serum (Sigma-Aldrich, MA, United States) and 1% penicillin-streptomycin (Thermo Scientific, MA, United States), followed by centrifugation at 1,500 rpm for 5 min at 4°C. Contaminating red blood cells (RBCs) were removed using ammonium chloride lysis buffer and centrifugation at 1,500 rpm for 5 min at 4°C. PBMCs were then frozen in a medium containing 10% dimethyl sulfoxide and stored in liquid nitrogen. Prior to use, cells were slowly thawed and centrifuged at 1,500 rpm for 5 min at 4°C, and then counted using a hemacytometer with trypan blue (Gibco, Thermo Scientific) to determine cell viability.

### PBMCs stimulation

PBMCs were cultured in a 96-well plate at a density of 5 × 10^5^ cells per well in 200 μL of RPMI 1,640 medium supplemented with 1X GlutaMAX, 10% human serum, and 1% penicillin/streptomycin. BKPyV antigen concentrations were optimized in pilot titrations (0.1, 0.5, 1, 2 μg/mL); only 1 μg/mL produced responses above background and 2 μg/mL did not improve signal, so this 1 μg/mL dose was used in all assays. The cells were subjected to eight different conditions to ensure unbiased stimulation results, including the negative control (unstimulated PBMCs), a positive control with phorbol myristate acetate (PMA) and ionomycin, stimulation with 1 μg/mL BKPyV LTA (PepTivator, Miltenyi Biotec, Bergisch Gladbach, Germany), stimulation with 1 μg/mL BKPyV VP1 (PepTivator, Miltenyi Biotec, Bergisch Gladbach, Germany), a combination of 0.5 μg/mL LTA and 0.5 μg/mL VP1, LTA combined with stimulatory antibodies against 1 μg/mL CD28 (eBioscience, CA, United States) and 1 μg/mL CD49d (eBioscience, CA, United States), VP1 combined with anti-CD28 and anti-CD49d, and a combination of LTA, VP1, anti-CD28, and anti-CD49d. The cells were incubated for 24 h in a humidified atmosphere with 5% CO_2_ at 37°C, and 4 h prior to the endpoint, a protein transport inhibitor cocktail (eBioscience, CA, United States) was added to the cell cultures.

### Staining for intracellular cytokines and surface markers of activated T-cells

After the incubation period, PBMCs were transferred to a 96-well V-bottom plate, washed twice with cold phosphate-buffered saline (PBS), and centrifuged at 1,500 rpm for 5 min at 4°C. The cells were then resuspended in FACS staining buffer (PBS containing 0.5% bovine serum albumin) and incubated with antibodies targeting CD3-APC (clone OKT3, BioLegend, CA, United States), CD4-Alexa Fluor 700 (clone RPA-T4, BioLegend, CA, United States), CD8a-FITC (clone RPA-T8, BioLegend, CA, United States), CD25-PerCP/Cyanine5.5 (clone BC96, BioLegend, CA, United States), CD134-Brilliant Violet 421 (clone Ber-ACT35, BioLegend, CA, United States), CD137-Brilliant Violet 605 (clone 4B4-1, BioLegend, CA, United States), and CD154-APC/Cy7 (clone 24–31, BioLegend, CA, United States) for 30 min in the dark. Following surface marker staining, the cells were washed with FACS staining buffer and fixed with fixation buffer (BioLegend, CA, United States) for 15 min in the dark. Next, the cells were washed twice with 1X intracellular staining permeabilization wash buffer (BioLegend, CA, United States) at 1,500 rpm for 10 min at 4°C, and then stained with antibodies against IFN-γ-PE (clone B27, BioLegend, CA, United States), IL-2-PE/Cy7 (clone MQ1-17H12, BioLegend, CA, United States), and TNF-α-Brilliant Violet 510 (clone MAb11, BioLegend, CA, United States) for 30 min in the dark. Data were captured using the BD FACSLyric™ flow cytometry system (BD Bioscience, NJ, United States).

### Flow cytometry analysis

PBMCs from all conditions for each participant were analyzed using Kaluza Analysis Software version 2.2 (Beckman Coulter, CA, United States). The negative control (PBMCs without any stimulation or BKPyV antigens) was used to establish the gating strategy, as illustrated in Supplementary Figure 1A. Percentages of positive cells—after background subtraction using the negative control—were extracted for IFN-γ, IL-2, TNF-α, CD25, CD134, CD137, and CD154 from CD3^+^ T cells, CD4^+^ T helper cells, and CD8^+^ cytotoxic T cells. These analyses were conducted to determine the overall T cell response (CD3^+^) and its subsets (CD4^+^ and CD8^+^), emphasizing their potential practical application. Boolean gating was used to analyze combinations of markers. Given the limited number of available channels on flow cytometer, live/dead staining (eBioscience, CA, United States) was performed prior to antibody staining and fixation/permeabilization using a separate aliquot from the same stimulation (Supplementary Figure 1B). Only samples with ≥ 90% viability were advanced to downstream staining. The raw, unsubtracted data for each surface marker and intracellular cytokine are presented in Supplementary Figures 2A–I.

### Statistical analysis

Continuous data are presented as the mean and standard deviation (SD) for normally distributed variables, and as the median and interquartile range (IQR) for non-parametric data. Comparisons between groups were performed using the *t*-test or the Wilcoxon Rank Sum test, as appropriate. To evaluate the cytokines and surface activation markers following BKPyV antigen stimulation, *p*-values for differences between groups (BK vs. nBK vs. HC) were calculated and displayed in heatmaps, with lighter colors indicating lower *p*-values. Candidate cytokines and surface activation markers with potential clinical utility were then analyzed using logistic regression for the diagnosis of BKPyV viremia, and the area under the receiver operating characteristic curve (AUROC) was calculated. Finally, the selected cytokines/markers were further analyzed to determine potential cutoffs, sensitivity, and specificity for clinical practice.

### Ethical considerations

This study was approved by the Institutional Review Board of the Faculty of Medicine, Chulalongkorn University, Bangkok, Thailand, and was conducted in accordance with international guidelines for human research protection, including the Declaration of Helsinki, the Belmont Report, CIOMS Guidelines, and the International Conference on Harmonization’s Good Clinical Practice standards (IRB No. 0167/66). Data used in the analyses were de-identified to ensure anonymity, guaranteeing that no participants could be identified. Furthermore, the clinical and research activities reported are consistent with the principles outlined in the Declaration of Istanbul on Organ Trafficking and Transplant Tourism.

## Results

The characteristics of the KTR and control groups are presented in Table 1. There were no significant differences between the KTR with BKPyV viremia (BK group) and those without BKPyV viremia (nBK group) in terms of age, sex, human leukocyte antigen (HLA) mismatches, time after transplantation, or immunosuppressive medications used at the time of blood collection. In the BK group, the median BKPyV viral load at specimen collection—corresponding to each patient’s first viremia—was 1,222 copies/mL (IQR 338–1,720).

**TABLE 1:** Characteristics of kidney transplant recipients with low-level BKPyV viremia (BK; *n* = 12), without BKPyV viremia (nBK; *n* = 12), and healthy control (HC; *n* = 12).

Variables	BK	nBK	*P*-value BK vs. nBK*	HC
Age, years (mean ± SD)	47.1 ± 8.1	48.3 ± 9.0	0.75	39.1 ± 5.6
Male, n (%)	9 (75%)	9 (75%)	1.00	9 (75%)
Dialysis vintage, years (mean ± SD)	4.7 ± 2.7	5.3 ± 2.4	0.59	–
HLA mismatch for A, B, DR	2.8 ± 1.2	2.3 ± 1.7	0.43	–
Deceased donor, n (%)	4 (33%)	3 (25%)	0.65	–
Basiliximab induction, n (%)	12 (100%)	12 (100%)	1.00	–
Time at sample collection, months after transplantation (median and IQR)	6 (3–13)	6 (5–9)	0.95	–
Tacrolimus C_0_, ng/mL (mean ± SD)	6.5 ± 1.4	6.4 ± 1.5	0.65	–
Mycophenolate mofetil dose, mg/day (mean ± SD)	1,125 ± 216	1,041 ± 138	0.34	–
Prednisolone dose, mg/day (mean ± SD)	5.6 ± 2.1	5.2 ± 1.6	0.61	–
Serum BK viral load at sample collection (first onset BKPyV viremia), copies/mL (median and IQR)	1,222 (338–1,720)	–	–	–
Serum creatinine, mg/dL (mean ± SD)	2.28 ± 1.13	1.55 ± 0.54	0.07	–

*P-values were calculated using *t*-test, Wilcoxon Rank Sum test (for non-parametric data), and chi-square test. HLA, human leukocyte antigen.

### Comparison of cytokines and activation markers between BK, nBK, and HC groups

**FIGURE 1:**
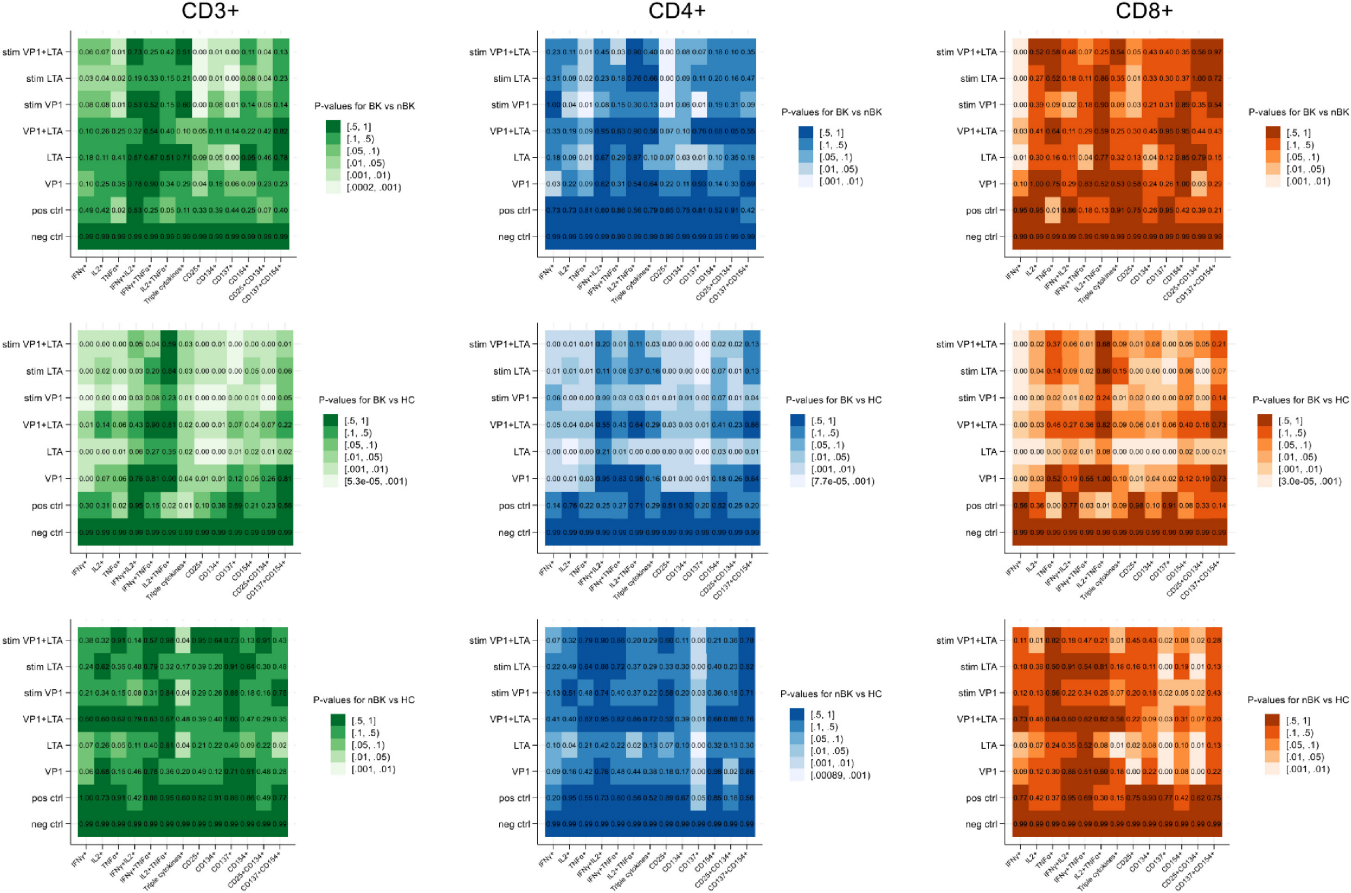
Heatmaps illustrating the *p*-values for differences in cell percentages of intracellular cytokine and surface marker expression following BKPyV antigen stimulation (Wilcoxon Rank Sum test). Patients with BKPyV viremia (BK) exhibit the lowest percentages, followed by those with non- BKPyV viremia (nBK) and healthy controls (HC). Lighter colors represent lower *p*-values. LTA, large T antigen; VP1, viral capsid protein 1 antigen; stim, co-stimulated with CD28 and CD49d antibodies.

Candidate cytokines and surface markers of activated T cells were first evaluated under various stimulation conditions. Figure 1 presents heatmaps of *p*-values for differences in the percentages of cells positive for intracellular cytokines (IFN-γ, IL-2, and TNF-α) and surface activation markers (CD25, CD134, CD137, and CD154) among CD3^+^ T cells, CD4^+^ T helper cells, and CD8^+^ cytotoxic T cells. These comparisons were made between BK versus nBK and HC, as well as between nBK and HC groups. The most pronounced differences were observed when comparing the BK and HC groups. Notably, for the clinically relevant comparison between BK and nBK groups, VP1 and/or LTA co-stimulated with CD28/CD49d yielded the highest discriminatory power across all T cell subsets.

**FIGURE 2:**
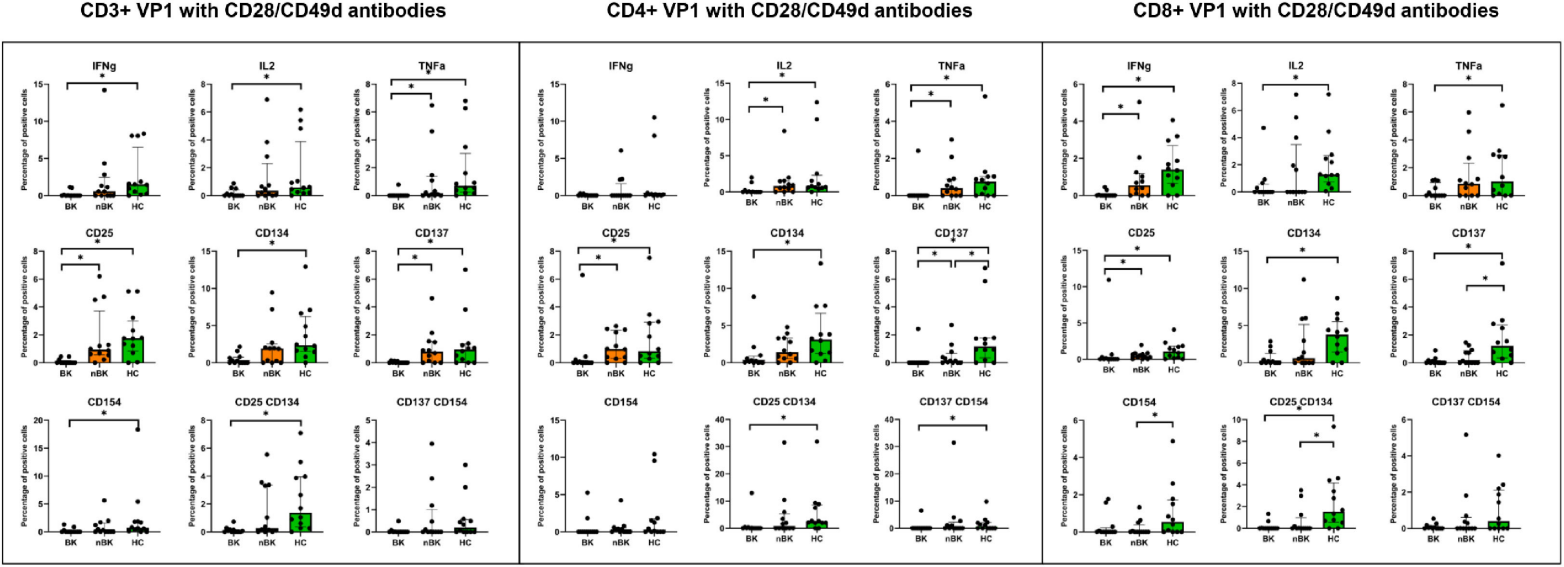
The percentage of positive cells—defined by the expression of intracellular cytokines and surface markers—was measured following stimulation with VP1 antigen and CD28/CD49d antibodies in CD3 + T cells, CD4 + helper T cells, and CD8 + cytotoxic T cells. Comparisons between groups were performed using the Wilcoxon Rank Sum test. *p-value <0.05.

**FIGURE 3:**
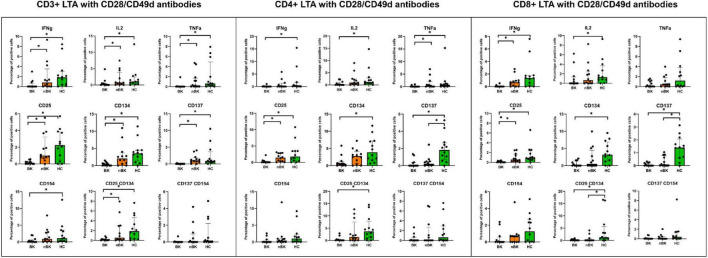
The percentage of positive cells—defined by the expression of intracellular cytokines and surface markers—was measured following stimulation with LTA antigen and CD28/CD49d antibodies in CD3 + T cells, CD4 + helper T cells, and CD8 + cytotoxic T cells. Comparisons between groups were performed using the Wilcoxon Rank Sum test. *p-value <0.05.

**FIGURE 4:**
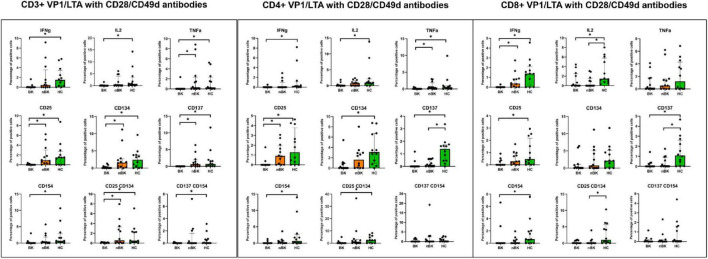
The percentage of positive cells—defined by the expression of intracellular cytokines and surface markers—was measured following stimulation with combined VP1 and LTA antigen and CD28/CD49d antibodies in CD3 + T cells, CD4 + helper T cells, and CD8 + cytotoxic T cells. Comparisons between groups were performed using the Wilcoxon Rank Sum test. *p-value <0.05.

Figures 2–4 illustrate the actual percentages of positive cells after stimulation with VP1, LTA, and the combination of VP1 and LTA, respectively. In these figures, cells were co-stimulated with CD28/CD49d because stimulation with BKPyV antigens alone (either VP1 or LTA) did not provide sufficient discriminatory data between the BK and nBK groups, as shown in Figure 1. Overall, the percentage of positive cells was highest in the HC group, followed by the nBK group, with the BK group exhibiting the lowest values. However, not all comparisons reached statistical significance. In VP1-stimulated cells, CD4^+^ T cells exhibited significant differences between the BK and nBK groups for IL-2, TNF-α, CD25, and CD137, whereas VP1-stimulated CD8^+^ T cells showed significant differences for IFN-γ and CD25. Similarly, for LTA stimulation in the BK versus nBK comparison, CD4^+^ T cells showed significant differences for TNF-α and CD25, while CD8^+^ T cells differed significantly for IFN-γ and CD25. T cells stimulated with the combination of VP1 and LTA demonstrated a pattern similar to that observed with the individual antigens. For potential clinical implementation, isolated VP1 and LTA—each co-stimulated with CD28/CD49d—were selected for further analysis via AUROC to diagnose BKPyV viremia.

To assess robustness, we conducted a sensitivity analysis using the stimulation index (SI), defined as the percentage of positive cells in the stimulated condition divided by that in the matched unstimulated condition. Supplementary Figure 3 presents a heatmap of *p*-values for differences in SI across the BK, nBK, and HC groups, showing a pattern of differences comparable to that obtained with the subtraction method.

### AUROC and candidate markers for clinical utility

Table 2 displays the AUROC values for intracellular cytokine and surface activation marker expression in CD4^+^ and CD8^+^ T cells. Significant AUROC values were observed for LTA-stimulated CD4^+^CD25^+^ T cells (0.823, 95%CI 0.657–0.989, *p* = 0.030), VP1-stimulated CD8^+^IFN-γ^+^ T cells (0.816, 95%CI 0.648–0.984, *p* = 0.045), and LTA-stimulated CD8^+^IFN-γ^+^ T cells (0.833, 95%CI 0.678–0.989, *p* = 0.028). Given that LTA stimulation provided good discrimination between the BK and nBK groups in both CD4^+^ and CD8^+^ T cells, LTA-stimulated CD4^+^CD25^+^ and CD8^+^IFN-γ^+^ T cells were chosen as candidate markers to further assess sensitivity, specificity, and an optimal cutoff.

**TABLE 2:** Area under the receiver operating characteristic curve (AUROC) values for intracellular cytokine and surface activation marker expression in CD4 + and CD8 + T cells following stimulation with VP1 or LTA (both co-stimulated with CD28/CD49d), for the diagnosis of BKPyV viremia in kidney transplant recipients compared to those without BKPyV viremia.

Cell	Stimulated antigen	Cytokine/marker	AUROC	*P*-value
**CD4 + T cells**	**VP1**	CD134	0.722 (0.497–0.947)	0.367
		CD137	0.771 (0.591–0.950)	0.370
		CD154	0.368 (0.166–0.570)	0.936
		CD25	0.799 (0.602–0.996)	0.356
		IFN-γ	0.500 (0.300–0.700)	0.292
		IL-2	0.733 (0.522–0.944)	0.111
		TNF-α	0.771 (0.591–0.950)	0.193
	**LTA**	CD134	0.701 (0.469–0.934)	0.071
		CD137	0.688 (0.461–0.914)	0.490
		CD154	0.646 (0.421–0.870)	0.429
		CD25	0.823 (0.657–0.989)	**0.030**
		IFN-γ	0.611 (0.393–0.829)	0.248
		IL-2	0.701 (0.483–0.920)	0.163
		TNF-α	0.747 (0.563–0.930)	0.226
**CD8 + T cells**	**VP1**	CD134	0.646 (0.424–0.868)	0.164
		CD137	0.611 (0.393–0.829)	0.119
		CD154	0.514 (0.310–0.718)	0.644
		CD25	0.260 (0.051–0.469)	0.619
		IFN-γ	0.816 (0.648–0.984)	**0.045**
		IL-2	0.590 (0.381–0.799)	0.203
		TNF-α	0.691 (0.481–0.901)	0.141
	**LTA**	CD134	0.611 (0.386–0.837)	0.161
		CD137	0.618 (0.392–0.844)	0.114
		CD154	0.601 (0.376–0.825)	0.491
		CD25	0.792 (0.593–0.990)	0.947
		IFN-γ	0.833 (0.678–0.989)	**0.028**
		IL-2	0.625 (0.401–0.849)	0.509
		TNF-α	0.580 (0.342–0.818)	0.300

Bold numerical *p*-values were <0.05.

Supplementary Table 1 reports the AUROC values from the SI-based analyses. Under VP1 stimulation, CD4^+^CD25^+^ and CD8^+^IFN-γ^+^ T cells demonstrated statistically significant discrimination. Under LTA stimulation, CD4^+^CD25^+^, CD8^+^CD25^+^, and CD8^+^IFN-γ^+^ T cells were significant. These findings are consistent with the subtraction-based analyses and support prioritizing LTA-stimulated CD4^+^CD25^+^ and CD8^+^IFN-γ^+^ T cells for subsequent analyses.

### Sensitivity, specificity, and AUROC of LTA/CD28/CD49d-stimulated CD4^+^CD25^+^ and CD8^+^IFN-γ^+^ T cells

**FIGURE 5:**
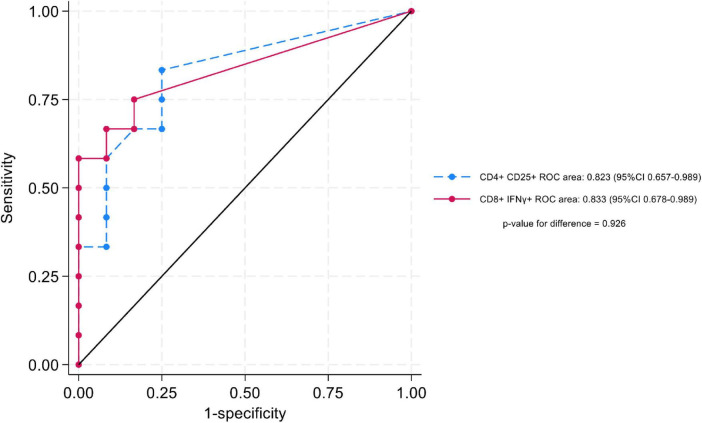
Area under the receiver operating characteristic curve (AUROC), comparing LTA/CD28/CD49d-stimulated CD4 + CD25 + and CD8 + IFN-γ + T cells.

A cutoff of > 0.2% positive cells (after background subtraction) for LTA/CD28/CD49d-stimulated CD4^+^CD25^+^ and CD8^+^IFN-γ^+^ T cells demonstrated the best sensitivity and specificity. Using this cutoff, 79.2% of KTR in the nBK group were correctly classified as having positive cell percentages > 0.2%, while 80.0% of KTR in the BK group were correctly classified as having positive cell percentages ≤ 0.2% (Table 3). Notably, none of the KTR with BKPyV viremia exhibited a positive cell percentage > 0.2% for both markers. Figure 5 depicts the AUROC for both stimulated cell populations, with no significant difference between them (*p* = 0.926).

**TABLE 3:** Sensitivity and specificity of LTA/CD28/CD49d-stimulated CD4 + CD25 + and CD8 + IFN-γ + T cells, using a cutoff of > 0.2% positive cells (after background subtraction) to diagnose KTR without BKPyV viremia.

Marker	Sensitivity (95%CI)	Specificity (95%CI)	Correctly classified	Positive predictive value (95%CI)	Negative predictive value (95%CI)
CD4 + CD25 + T cells	76.9 (46.2–95.0)%	81.8 (48.2–97.7)%	79.2%	83.3 (51.6–97.9)%	75.0 (42.8–94.5)%
CD8 + IFN-γ + T cells	80.0 (44.4–97.5)%	71.4 (41.9–91.6)%	75.0%	66.7 (34.9–90.1)%	83.3 (51.6–97.9)%

None of the KTR with BKPyV viremia exhibited a positive cell percentage > 0.2% for both markers.

In the SI-based sensitivity analysis, a cutoff for the stimulated-to-unstimulated ratio > 1.2 for both LTA/CD28/CD49d-stimulated CD4^+^CD25^+^ and CD8^+^IFN-γ^+^ T cells yielded the optimal combination of sensitivity and specificity, as well as the highest percentage correctly classified for BKPyV viremia (Supplementary Table 2).

## Discussion

This study is the first to comprehensively analyze the BKPyV-specific cellular immune response in KTR with and without BKPyV viremia, using healthy controls as a reference. We evaluated both the cytokine responses and surface antigen markers of activated T cells. Notably, stimulation with BKPyV LTA in combination with CD28/CD49d co-stimulatory antibodies emerged as a promising approach for clinical application, eliciting robust responses in both CD4^+^ and CD8^+^ T cells. Among the biomarkers examined, CD4^+^CD25^+^ and CD8^+^IFN-γ^+^ T cells demonstrated sufficient discriminatory power to differentiate KTR with BKPyV viremia from those without. A cutoff value of > 0.2% positive cells (after background subtraction) yielded the highest rates of correct classification for both groups. By including only KTRs with low-level BKPyV viremia (<3,000 copies/mL) in the BK group, the observed differences in these biomarkers could serve as potential screening tools for the early detection of BKPyV reactivation—possibly even before the onset of detectable viremia—and could help guide timely adjustments to immunosuppressive therapy. However, this hypothesis is needed to be tested in the future cohort.

BKPyV infection is a major cause of kidney allograft dysfunction and is associated with significantly reduced allograft survival. A critical challenge in managing BKPyV infection after transplantation is the absence of effective antiviral treatments, as current strategies rely primarily on reducing immunosuppression. Consequently, preventing BKPyV infection is of paramount importance. Although previous studies have examined the cellular immune response to BKPyV post-transplantation, none have concurrently evaluated both intracellular cytokine production and surface activation marker expression (10–17, 24–35). Thus, a comprehensive assessment of the cellular immune response, particularly comparing transplant recipients with and without BKPyV infection, has not previously been undertaken. In an effort to identify the most appropriate immunological assay for BKPyV infection, we first explored the immune profiles that most effectively distinguish KTR with low-level BKPyV viremia from those without, laying the groundwork for a future clinical screening tool.

Cytokines such as IFN-γ, IL-2, and TNF-α have been implicated in the immune response to BKPyV infection in KTR (17, 36). IL-2, one of the earliest cytokines identified, is primarily produced by T cells and plays a central role in promoting T cell activation and proliferation, as well as regulating immune responses through its effects on regulatory T cells (37, 38). TNF-α acts as a frontline cytokine during viral infections and is produced by various cell types, notably macrophages and T cells (39–41). IFN-γ is essential for antiviral defense and for mediating the cytotoxic effects of CD8^+^ T cells, in addition to enhancing the function of other inflammatory cells such as macrophages, dendritic cells, and natural killer cells to prolong the antiviral state and strengthen the overall immune response during active infection (42–45). Collectively, these cytokines represent a crucial early immune response against BKPyV infection. Our study demonstrated that IFN-γ-producing CD8^+^ T cells provided effective differentiation between KTR with and without BKPyV viremia, highlighting the key role of CD8^+^ T cells in targeting and eliminating virally infected cells. However, cytokine analysis alone may not capture the full complexity of cellular immune responses, as T cells can be activated through multiple distinct pathways. This limitation reduces the accuracy of cytokine-based assays, such as ELISPOT, in assessing the overall immune response to BKPyV infection. Therefore, we also evaluated T-cell activation through surface marker expression, specifically CD25, CD134, CD137, and CD154.

CD25, the IL-2 receptor expressed on effector and regulatory T cells, serves as a marker of activation following antigen stimulation (46). CD134 (OX40) is a co-stimulatory molecule that sustains T cell responses, thereby preventing excessive viral replication (47, 48). Previous studies have demonstrated that antigen-specific CD4^+^ T cells co-expressing CD25 and CD134 can be detected at substantially higher levels compared to intracellular cytokine assays, highlighting their potential diagnostic value (20). Although our findings revealed significant differences in CD25^+^CD134^+^CD4^+^ T cells between BK and HC groups, no significant difference was observed between BK and nBK groups, limiting the clinical utility of this co-expression marker for distinguishing BKPyV infection in KTR. However, CD4^+^CD25^+^ T cells alone demonstrated adequate discriminatory power between BK and nBK groups, underscoring their potential as a diagnostic marker. In our cohort, LTA-stimulated CD4^+^CD25^+^ T cells were significantly reduced in KTRs with early BKPyV viremia compared with aviremic controls. Since tacrolimus inhibits IL-2 production, we propose that this CD25 (IL-2 receptor α-chain) suppression may be related to tacrolimus exposure, consistent with reports linking tacrolimus use to increased BKPyV risk (49). Although whole-blood tacrolimus troughs were comparable between groups in this study (Table 1), whole-blood measurements largely reflect drug bound to erythrocytes and plasma proteins (99%), whereas only a small free/intracellular fraction is pharmacologically active (approximately 1%) (50, 51). Intracellular tacrolimus is not routinely measured because of technical constraints, yet evidence suggests that intracellular levels correlate more closely with both anti-rejection efficacy and toxicity than total whole-blood concentrations (50, 51). Accordingly, a functional readout, such as suppression of LTA-stimulated CD4^+^CD25^+^ T cells, may better capture net calcineurin-inhibition *in vivo* and, therefore, relate more directly to BKPyV viremia. The MPA data available in this study consisted only of prescribed dose; no MPA concentration measurements were obtained. Future studies should include precise therapeutic drug monitoring to evaluate how immunosuppressive exposure influences BKPyV-specific cellular immunity markers.

CD137, an inducible co-stimulatory molecule belonging to the TNF receptor superfamily, enhances the antigen-specific response of both CD4^+^ and CD8^+^ T cells (19, 52). CD154 (also known as CD40L) is a marker of activated CD4^+^ T helper cells, playing a critical role in initiating humoral and effective cytotoxic responses (18, 53). The detection of increased CD137^+^CD154^+^ T cells improves the sensitivity of assessing low-frequency T-cell responses and correlates with intracellular cytokine production (23). In our analysis, both CD137 and CD154 expression showed significant differences between BK and HC groups; however, these differences were less pronounced when comparing the BK and nBK groups.

VP1 is a viral capsid protein and serves as one of the major structural proteins of the BKPyV, defining the four primary VP1 serotypes originally identified through neutralizing antibodies (54). LTA, on the other hand, is a multifunctional protein essential for viral replication and cell transformation, playing a critical role in viral oncogenesis by inhibiting the tumor suppressor protein p53 within the nucleus (55, 56). While VP1 has been predominantly associated with the humoral immune response, LTA is recognized as a key antigen for cytotoxic T-cell responses (54). This notion is supported by our study, which found that LTA more effectively elicited a cellular immune response capable of differentiating between BK and nBK groups in both CD4^+^ and CD8^+^ T cells. Further investigation into these differences in cellular immune activation is necessary, especially in larger populations, as previous studies have demonstrated that both VP1 (and VP3) and LTA stimulate cellular immune responses, although these studies primarily evaluated cytokine production without including surface activation markers (14, 29).

We analyzed multiple combinations of intracellular cytokines and surface activation markers expressed by activated T cells. Our findings revealed the most pronounced differences between the BK and HC groups, consistent with the immunological expectation that KTR with BKPyV infection exhibit a more suppressed immune response compared to healthy individuals. However, when comparing BK with non-viremic (nBK) recipients, fewer markers remained significantly different. Markers stimulated by either LTA or VP1 in combination with CD28 and CD49d co-stimulatory antibodies were selected for further analysis, given their practical utility and cost-effectiveness for clinical diagnostic laboratories. Notably, our results indicated that combining both antigens did not provide additional discriminatory power compared to using either antigen alone. The AUROC analyses demonstrated that LTA-stimulated CD4^+^CD25^+^ and CD8^+^IFN-γ^+^ T cells could effectively differentiate between BK and nBK groups, whereas VP1 stimulation was effective only for CD8^+^IFN-γ^+^ T cells. To illustrate the potential clinical utility of these findings, we proposed a practical cutoff of > 0.2% positive cells (after background subtraction using an unstimulated negative control). Although we acknowledge that these results are based on the diagnosis of BKPyV viremia rather than predicting BKPyV infection in KTR who have not yet developed the condition, this study successfully identified and selected potential biomarkers by contrasting BK and nBK groups, using HC as a biological reference. Furthermore, we provided detailed methodology and clearly defined costimulatory molecules, which were not thoroughly addressed in previous studies. Based on this information, these biomarkers will be further evaluated as potential screening tools for the early detection of BKPyV infection or reactivation in future study. For example, KTR who exhibit a percentage of LTA-stimulated CD4^+^CD25^+^ and CD8^+^IFN-γ^+^ T cells below a predefined cutoff during post-transplant screening may be at increased risk of developing BKPyV viremia or BKPyVAN. In such cases, early immunosuppression reduction could be considered as a preventive strategy before the onset of detectable viremia.

The strengths of this study include its comprehensive approach, minimizing the bias associated with selecting only preferred cytokines or surface activation markers. This unbiased analysis provides a more complete perspective on the cellular immune response compared to previously utilized BKPyV-specific ELISPOT assays or flow cytometric analyses. Additionally, various antigen stimulation methods were systematically evaluated to identify the most cost-effective protocol suitable for implementation in clinical diagnostic laboratories. Furthermore, we proposed practical cutoff values to facilitate initial clinical use.

This study represents the first step in a staged program to determine whether candidate cellular immune markers are associated with the clinically relevant early BKPyV viremia. To maximize biological contrast while minimizing confounding, the analysis focused on KTRs with low-level BKPyV viremia, used as a proxy for very early infection, and on controls who never developed viremia. Within this framework, LTA-stimulated CD4^+^CD25^+^ T cells and CD8^+^IFN-γ^+^ T cells differed significantly between KTRs with early BKPyV viremia and those without, even in a limited sample, they are strong candidates for evaluation in larger studies. We hypothesize that they may decline before viremia becomes detectable; accordingly, they will be prioritized for prospective validation despite the modest size of this initial study.

The next phase will be a longitudinal cohort of KTRs sampled at 3-, 6-, and 12-months post-transplant to assess whether these markers decrease prior to the onset of BKPyV viremia, including among currently aviremic recipients. Contingent on validation, an interventional study will test whether biomarker-guided immunosuppression adjustments can reduce subsequent BKPyV replication and related complications. Factors associated with this cellular immunity suppression, including the different induction and maintenance immunosuppression regimens, shall be studied in a larger cohort study. A potential downside of reducing immunosuppression on the basis of biomarkers is false-positive results, which can lead to unwarranted tapering and thereby elevate the risk of allograft rejection. The net clinical benefit of biomarker-guided tapering should be tested in interventional trials with graft survival and acute rejection as primary endpoints.

Our study, however, has several limitations. First, the sample size was relatively small. While our findings require validation in a larger population, the extensive and unbiased assessment of multiple cytokines and activation markers across different stimulation conditions likely identified the most robust markers. Although a larger-scale study may uncover additional significant markers, we believe the markers identified in this study will remain relevant. Second, the cross-sectional design only demonstrates an association between the identified markers and peak BKPyV viremia; their predictive capabilities still require evaluation. Future studies should investigate marker kinetics over time after transplantation, their correlation with immunosuppressive drug dosages or concentrations, and their responsiveness to adjustments in immunosuppression for BKPyV management. The proposed cutoff of > 0.2% positive cells remains to be confirmed. Although technical variability in flow cytometry may affect these values, we believe that the detailed methods provided here will enable other transplant centers to replicate the experiment and validate our findings. Finally, BKPyV genotype data were unavailable in this mechanistic study. Donor–recipient genotype mismatch may influence immune responses and should be evaluated in future cohort studies, with parallel assessment of cellular and humoral immunity.

Recent evidence suggests that the humoral immune response also plays a significant role in controlling BKPyV infection post-transplantation (57), warranting investigation alongside cellular immunity to encompass all aspects of the immune response, particularly regarding differences among BKPyV serotypes. Early evidence indicates that humoral immunity to BKPyV, measured as BKPyV-specific IgG, is not protective against viremia or BKPyV-associated nephropathy; rather, IgG levels appear to reflect infection intensity (58). However, a pre-transplant donor–recipient IgG mismatch, characterized by high donor and low recipient titers, predicts post-transplant viremia (59). More recent work shows that low levels of pre-transplant donor BKPyV genotype–specific neutralizing antibody in the recipient best predict BKPyV viremia risk (60, 61). Because neutralizing-antibody assays are technically complex, future studies should integrate these measures with cellular immunity readouts to improve risk stratification.

## Conclusion

In conclusion, the BKPyV-specific T-cell response has been comprehensively characterized using various stimulation methods. LTA stimulation combined with CD28/CD49d antibodies demonstrated the highest discriminatory capability between kidney transplant recipients with and without BKPyV viremia, specifically through the measurement of CD4^+^CD25^+^ and CD8^+^IFN-γ^+^ T cells responses. A proposed cutoff of > 0.2% positive cells was associated with adequate sensitivity and specificity, supporting its potential clinical utility.

## Data Availability

The datasets presented in this article are not readily available because the dataset is available upon reasonable request to corresponding author. Requests to access the datasets should be directed to suwasin.u@gmail.com.
