# Supplementary material for: Suppression of large t antigen-stimulated CD4+CD25+ and CD8+IFN-γ+ T cells is strongly associated with low level BK viremia in kidney transplant recipients
Source: Front Med (Lausanne). 2025 Nov 14;12:1662833. doi: 10.3389/fmed.2025.1662833 (PMC12691454; doi:10.3389/fmed.2025.1662833)

SUPPRESSION OF LARGE T ANTIGEN-STIMULATED $CD4^+CD25^+$ AND $CD8^+IFN\text{-}\gamma^+$ T CELLS IS STRONGLY ASSOCIATED WITH BK VIREMIA IN KIDNEY TRANSPLANT RECIPIENTS

Wilasinee Saisorn[1], Thunyatorn Wuttiputhanun[2,3,4], Jakapat Vanichanan1[5], Kamonwan Jutivorakool[5], Natavudh Townamchai[2,3,4], Yingyos Avihingsanon[2,3,4], Asada Leelahavanichkul[1,6], Suwasin Udomkarnjananun[1,2,3,4]

[1]Center of Excellence on Translational Research in Inflammation and Immunology (CETRII), Department of Microbiology, Faculty of Medicine, Chulalongkorn University, Bangkok, Thailand

[2]Division of Nephrology, Department of Medicine, Faculty of Medicine, Chulalongkorn University and King Chulalongkorn Memorial Hospital, The Thai Red Cross Society, Bangkok, Thailand

[3]Excellence Center for Organ Transplantation (ECOT), King Chulalongkorn Memorial Hospital, The Thai Red Cross Society, Bangkok, Thailand

[4]Renal Immunology and Renal Transplant Center of Excellence, Faculty of Medicine, Chulalongkorn University, Bangkok, Thailand

[5]Division of Infectious Disease, Department of Medicine, Faculty of Medicine, Chulalongkorn University and King Chulalongkorn Memorial Hospital, The Thai Red Cross Society, Bangkok, Thailand

Thai Transplantation Society

[6]Immunology Unit, Department of Microbiology, Chulalongkorn University, Bangkok, Thailand

Corresponding author

Suwasin Udomkarnjananun, MD, Ph.D.

Division of Nephrology, Department of Medicine, Faculty of Medicine, Chulalongkorn University and King Chulalongkorn Memorial Hospital, The Thai Red Cross Society, Bangkok, Thailand

Tel: +662-256-4251

Email: suwasin.u@gmail.com

Supplementary Table S1: Area Under the Receiver Operating Characteristic Curve (AUROC) values for intracellular cytokine and surface activation marker expression in CD4+ and CD8+ T cells following stimulation with VP1 or LTA (both co-stimulated with CD28/CD49d), for the diagnosis of BKPyV viremia in kidney transplant recipients compared to those without BKPyV viremia, using stimulated-to-unstimulated ratio.

Cell	Stimulated antigen	Cytokine/marker	AUROC	p-value
**CD4+ T cells**	**VP1**	CD134	0.694 (0.475-0.914)	0.238
		CD137	0.826 (0.649-1.000)	0.185
		CD154	0.313 (0.084-0.541)	0.970
		CD25	0.813 (0.626-0.999)	**0.033**
		IFN-γ	0.694 (0.468-0.921)	0.342
		IL-2	0.729 (0.504-0.954)	0.305
		TNF-α	0.833 (0.622-1.000)	0.052
	**LTA**	CD134	0.708 (0.486-0.930)	0.111
		CD137	0.729 (0.508-0.951)	0.137
		CD154	0.708 (0.491-0.926)	0.172
		CD25	0.819 (0.646-0.993)	**0.035**
		IFN-γ	0.729 (0.522-0.936)	0.127
		IL-2	0.694 (0.466-0.923)	0.242
		TNF-α	0.833 (0.670-0.997)	**0.037**
**CD8+ T cells**	**VP1**	CD134	0.674 (0.440-0.907)	0.091
		CD137	0.604 (0.350-0.859)	0.083
		CD154	0.521 (0.277-0.765)	0.775
		CD25	0.729 (0.506-0.952)	0.058
		IFN-γ	0.764 (0.543-0.985)	**0.032**
		IL-2	0.486 (0.229-0.743)	0.624
		TNF-α	0.681 (0.438-0.923)	0.103
	**LTA**	CD134	0.694 (0.470-0.919)	0.058
		CD137	0.694 (0.472-0.917)	0.091
		CD154	0.590 (0.342-0.838)	0.432
		CD25	0.847 (0.681-1.000)	**0.032**
		IFN-γ	0.785 (0.581-0.989)	**0.038**
		IL-2	0.597 (0.346-0.849)	0.246
		TNF-α	0.681 (0.455-0.906)	0.153

Bold; p-value < 0.05

Supplementary Table S2: Sensitivity and specificity of LTA/CD28/CD49d-stimulated CD4+CD25+ and CD8+IFN-γ+ T cells, using a stimulated-to-unstimulated ratio >1.2 as the cutoff to diagnose KTR without BKPyV viremia.

**Marker**	**Sensitivity (95%CI)**	**Specificity (95%CI)**	**Correctly classified**	**Positive predictive value (95%CI)**	**Negative predictive value (95%CI)**
CD4+CD25+ T cells	76.9 (46.2-95.0) %	81.8 (48.2-97.7) %	79.2 %	83.3 (51.6-97.9) %	75.0 (42.8-94.5) %
CD8+IFN-γ+ T cells	90.0 (55.5-99.7) %	78.6 (49.2-95.3) %	79.2 %	75.0 (42.8-94.5) %	91.7 (61.5-99.8) %

Supplementary Figure S1: Gating strategy for flow cytometry: (A) Gates were defined using the negative control (unstimulated sample). (B) Live/dead staining was performed prior to antibody staining and fixation/permeabilization, using a separate aliquot.

A

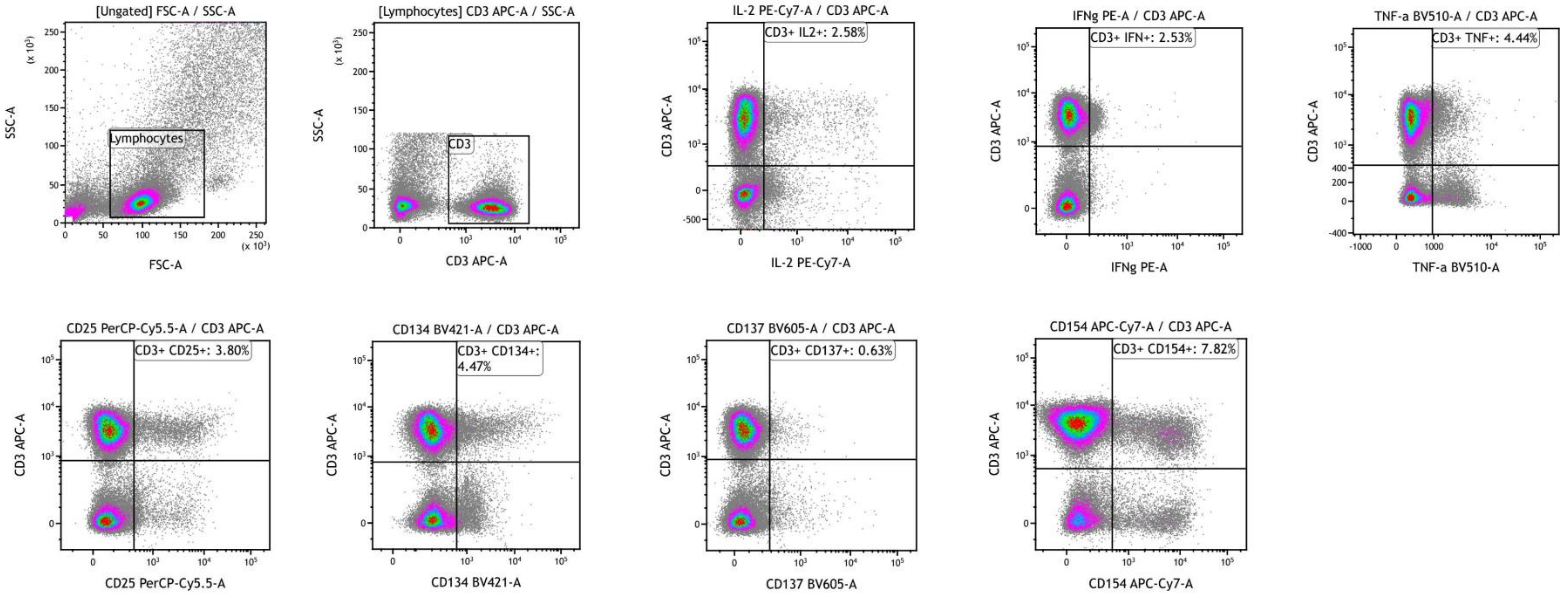

B

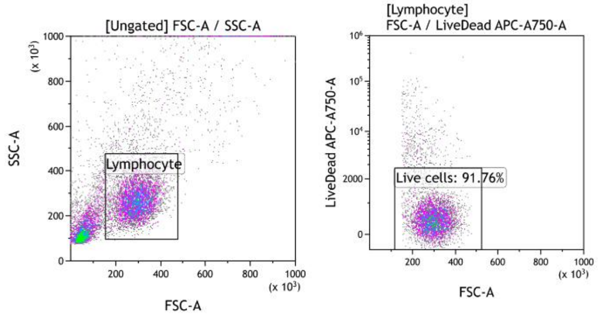

Supplementary Figure S2: Raw data for each surface marker and intracellular cytokine, presented as boxplots with median values stratified by cell type and stimulation condition. (A) IL-2 (B) IFN-γ (C) TNF-α (D) CD25 (E) CD134 (F) CD137 (G) CD154 (H) CD25/CD134 (I) CD137/CD154

A

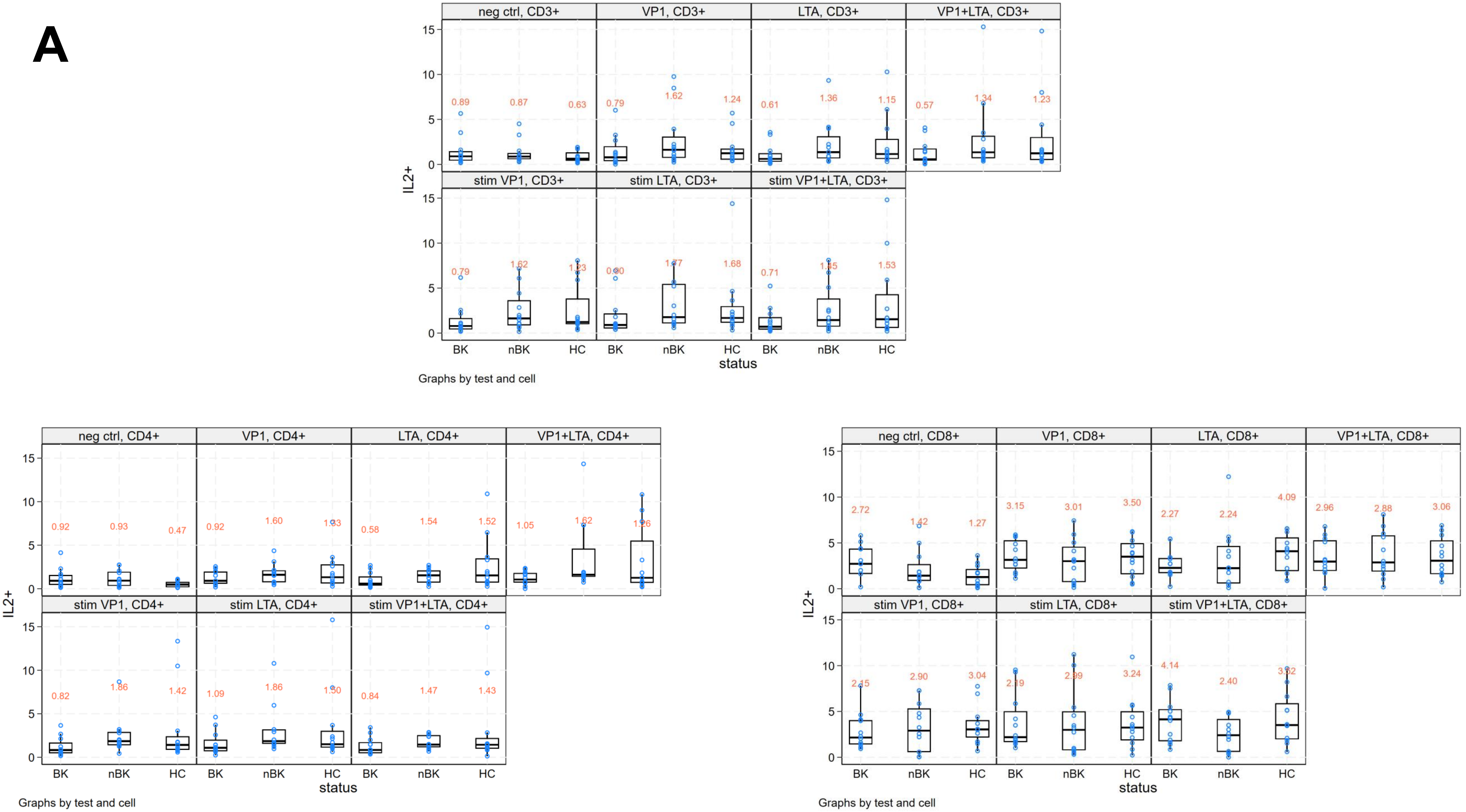
neg ctrl, CD3+
VP1, CD3+
LTA, CD3+
VP1+LTA, CD3+
stim VP1, CD3+
stim LTA, CD3+
stim VP1+LTA, CD3+
0.89 0.87 0.63
0.79 1.62 1.24
0.61 1.36 1.15
0.57 1.34 1.23
0.79 1.62 1.23
0.90 1.77 1.68
0.71 1.45 1.53
IL2+
0 5 10 15
BK nBK HC
status
Graphs by test and cell
neg ctrl, CD4+
VP1, CD4+
LTA, CD4+
VP1+LTA, CD4+
stim VP1, CD4+
stim LTA, CD4+
stim VP1+LTA, CD4+
0.92 0.93 0.47
0.92 1.60 1.53
0.58 1.54 1.52
1.05 1.62 1.26
0.82 1.86 1.42
1.09 1.86 1.50
0.84 1.47 1.43
IL2+
0 5 10 15
BK nBK HC
status
Graphs by test and cell
neg ctrl, CD8+
VP1, CD8+
LTA, CD8+
VP1+LTA, CD8+
stim VP1, CD8+
stim LTA, CD8+
stim VP1+LTA, CD8+
2.72 1.42 1.27
3.15 3.01 3.50
2.27 2.24 4.09
2.96 2.88 3.06
2.15 2.90 3.04
2.19 2.99 3.24
4.14 2.40 3.52
IL2+
0 5 10 15
BK nBK HC
status
Graphs by test and cell

B

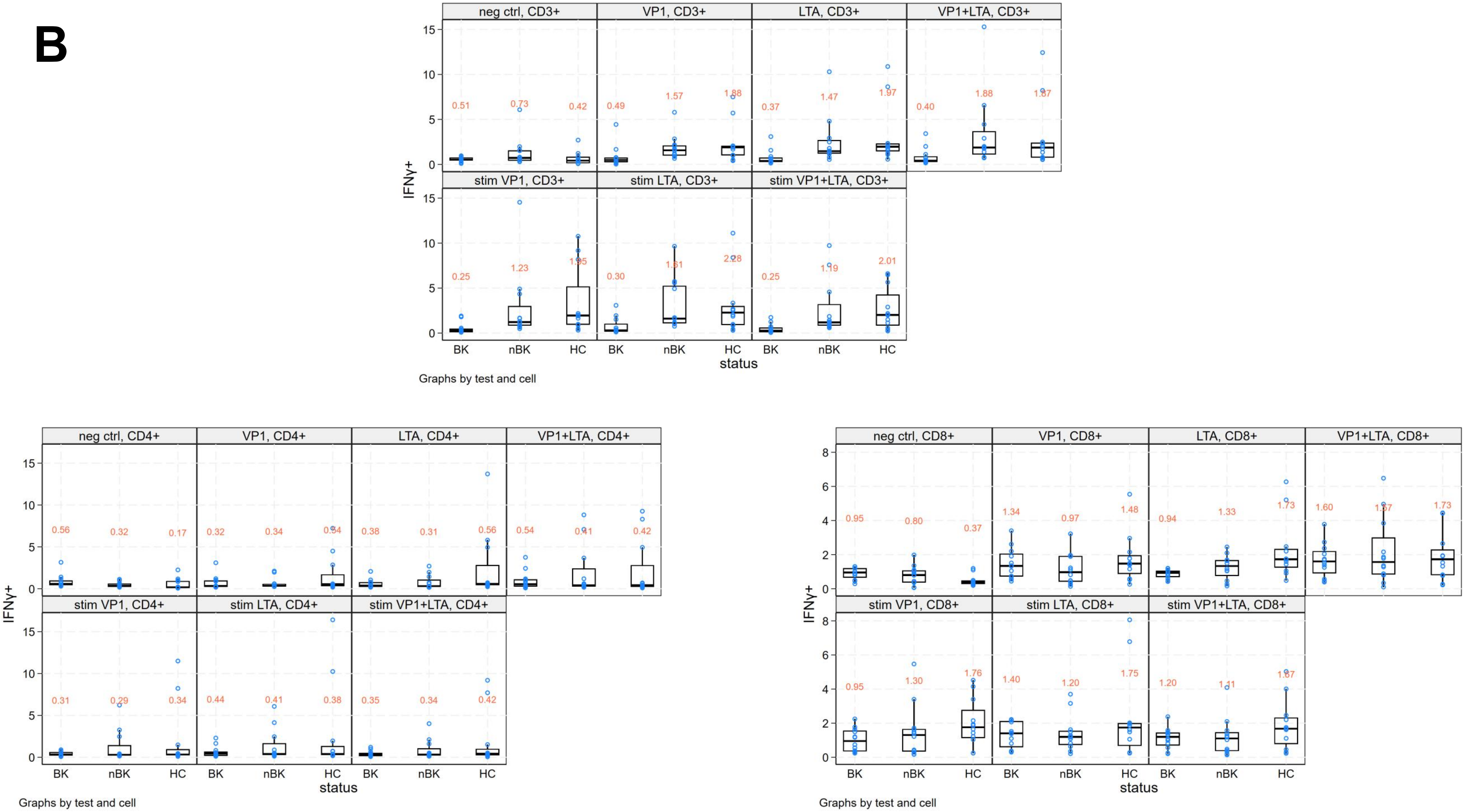

neg ctrl, CD3+
VP1, CD3+
LTA, CD3+
VP1+LTA, CD3+
stim VP1, CD3+
stim LTA, CD3+
stim VP1+LTA, CD3+
0.51 0.73 0.42
0.49 1.57 1.88
0.37 1.47 1.97
0.40 1.88 1.87
0.25 1.23 1.95
0.30 1.51 2.28
0.25 1.19 2.01
IFNγ+
BK nBK HC
status
Graphs by test and cell
neg ctrl, CD4+
VP1, CD4+
LTA, CD4+
VP1+LTA, CD4+
stim VP1, CD4+
stim LTA, CD4+
stim VP1+LTA, CD4+
0.56 0.32 0.17
0.32 0.34 0.54
0.38 0.31 0.56
0.54 0.41 0.42
0.31 0.29 0.34
0.44 0.41 0.38
0.35 0.34 0.42
IFNγ+
BK nBK HC
status
Graphs by test and cell
neg ctrl, CD8+
VP1, CD8+
LTA, CD8+
VP1+LTA, CD8+
stim VP1, CD8+
stim LTA, CD8+
stim VP1+LTA, CD8+
0.95 0.80 0.37
1.34 0.97 1.48
0.94 1.33 1.73
1.60 1.57 1.73
0.95 1.30 1.76
1.40 1.20 1.75
1.20 1.11 1.67
IFNγ+
BK nBK HC
status
Graphs by test and cell

C

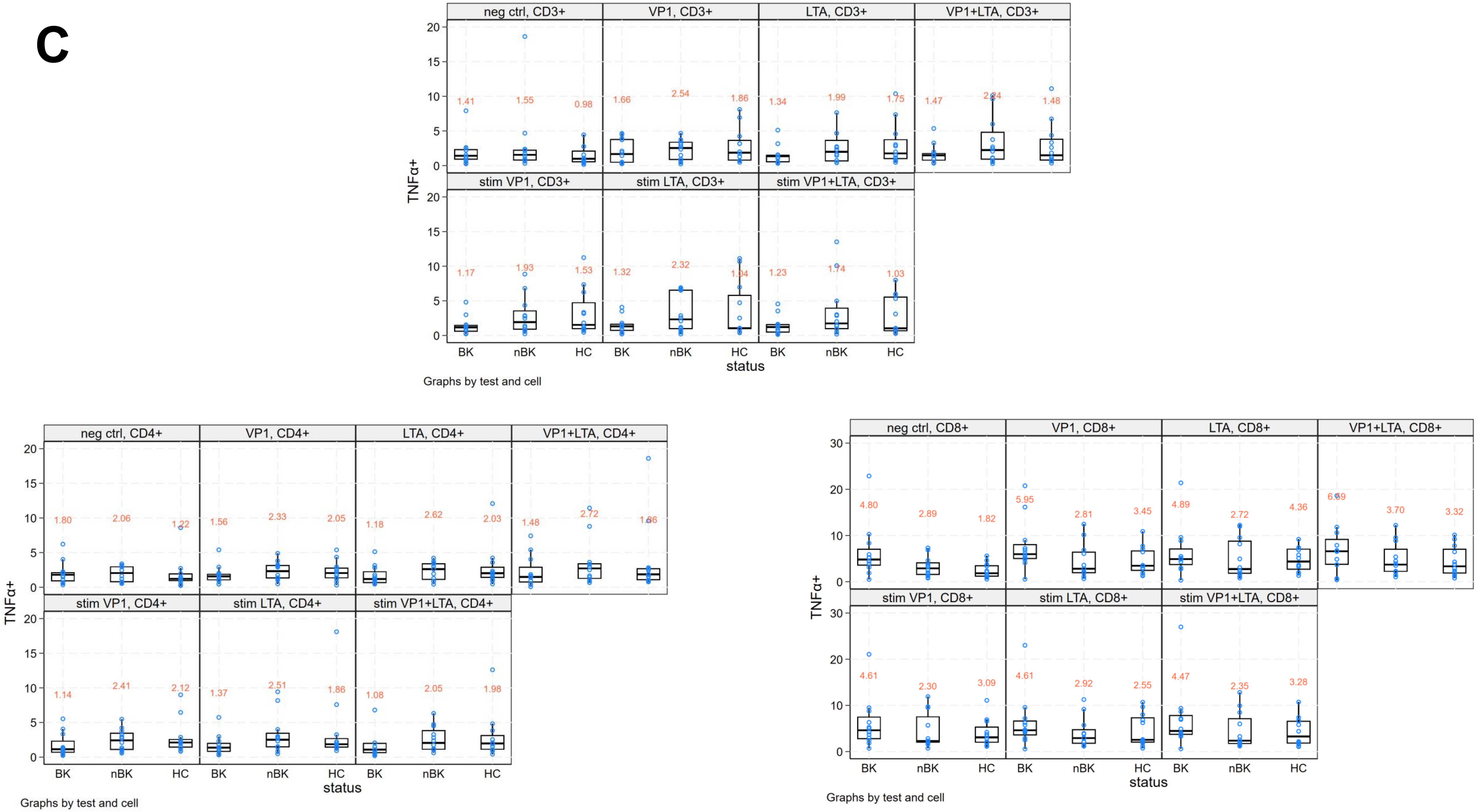
neg ctrl, CD3+
VP1, CD3+
LTA, CD3+
VP1+LTA, CD3+
stim VP1, CD3+
stim LTA, CD3+
stim VP1+LTA, CD3+
TNFα+
status
BK
nBK
HC
1.41
1.55
0.98
1.66
2.54
1.86
1.34
1.99
1.75
1.47
1.48
1.17
1.93
1.53
1.32
2.32
1.23
1.03
Graphs by test and cell
neg ctrl, CD4+
VP1, CD4+
LTA, CD4+
VP1+LTA, CD4+
stim VP1, CD4+
stim LTA, CD4+
stim VP1+LTA, CD4+
1.80
2.06
1.22
1.56
2.33
2.05
1.18
2.62
2.03
1.48
2.72
1.14
2.41
2.12
1.37
2.51
1.86
1.08
2.05
1.98
neg ctrl, CD8+
VP1, CD8+
LTA, CD8+
VP1+LTA, CD8+
stim VP1, CD8+
stim LTA, CD8+
stim VP1+LTA, CD8+
4.80
2.89
1.82
5.95
2.81
3.45
4.89
2.72
4.36
3.70
3.32
4.61
2.30
3.09
4.61
2.92
2.55
4.47
2.35
3.28

D

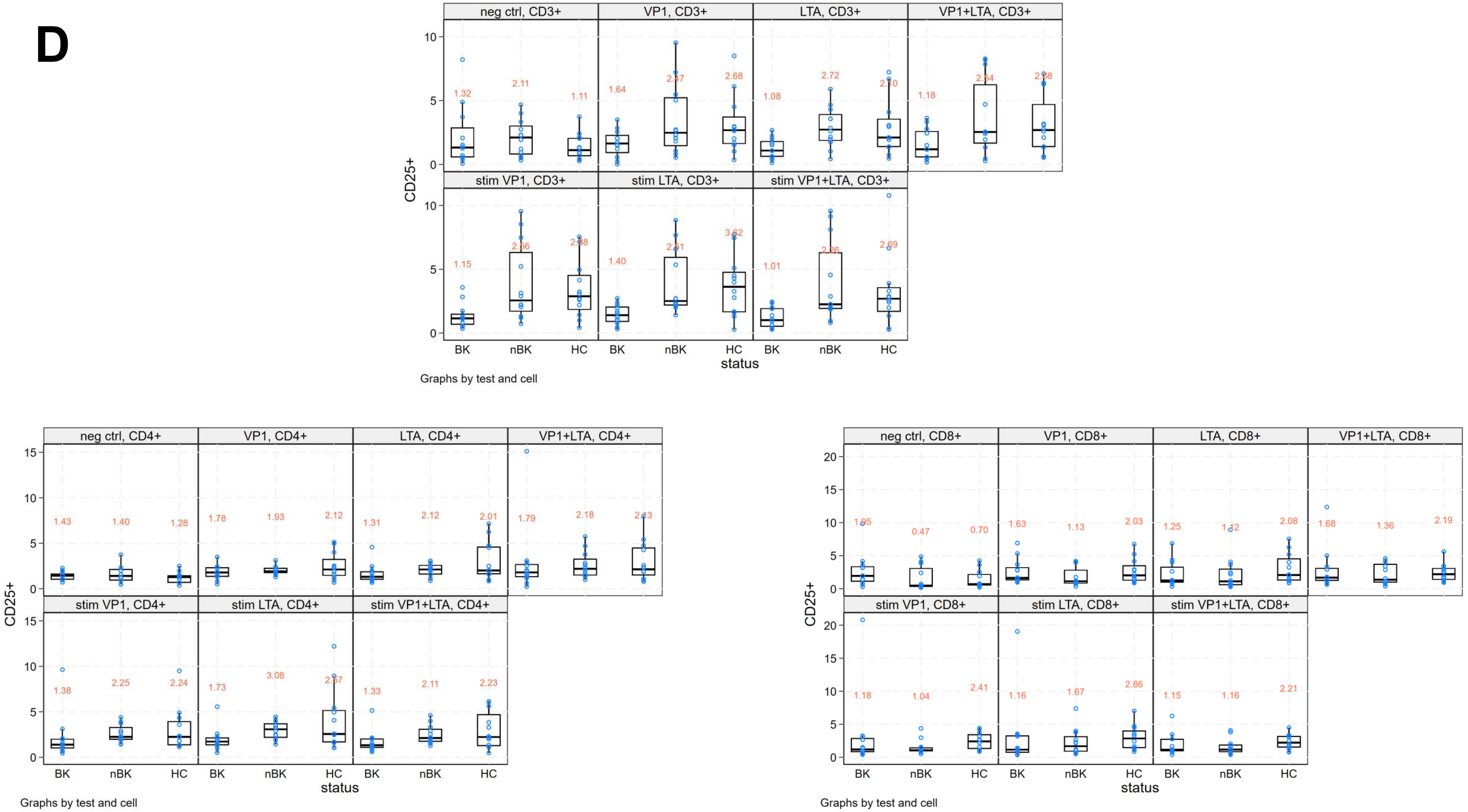
neg ctrl, CD3+
VP1, CD3+
LTA, CD3+
VP1+LTA, CD3+
stim VP1, CD3+
stim LTA, CD3+
stim VP1+LTA, CD3+
1.32
2.11
1.11
1.64
2.47
2.68
1.08
2.72
2.10
1.18
2.54
2.58
1.15
2.56
2.68
1.40
2.51
3.62
1.01
2.26
2.69
CD25+
status
BK
nBK
HC
Graphs by test and cell
neg ctrl, CD4+
VP1, CD4+
LTA, CD4+
VP1+LTA, CD4+
stim VP1, CD4+
stim LTA, CD4+
stim VP1+LTA, CD4+
1.43
1.40
1.28
1.78
1.93
2.12
1.31
2.12
2.01
1.79
2.18
2.43
1.38
2.25
2.24
1.73
3.08
2.57
1.33
2.11
2.23
CD25+
status
Graphs by test and cell
neg ctrl, CD8+
VP1, CD8+
LTA, CD8+
VP1+LTA, CD8+
stim VP1, CD8+
stim LTA, CD8+
stim VP1+LTA, CD8+
1.95
0.47
0.70
1.63
1.13
2.03
1.25
1.12
2.08
1.68
1.36
2.19
1.18
1.04
2.41
1.16
1.67
2.86
1.15
1.16
2.21
CD25+
status
Graphs by test and cell

E

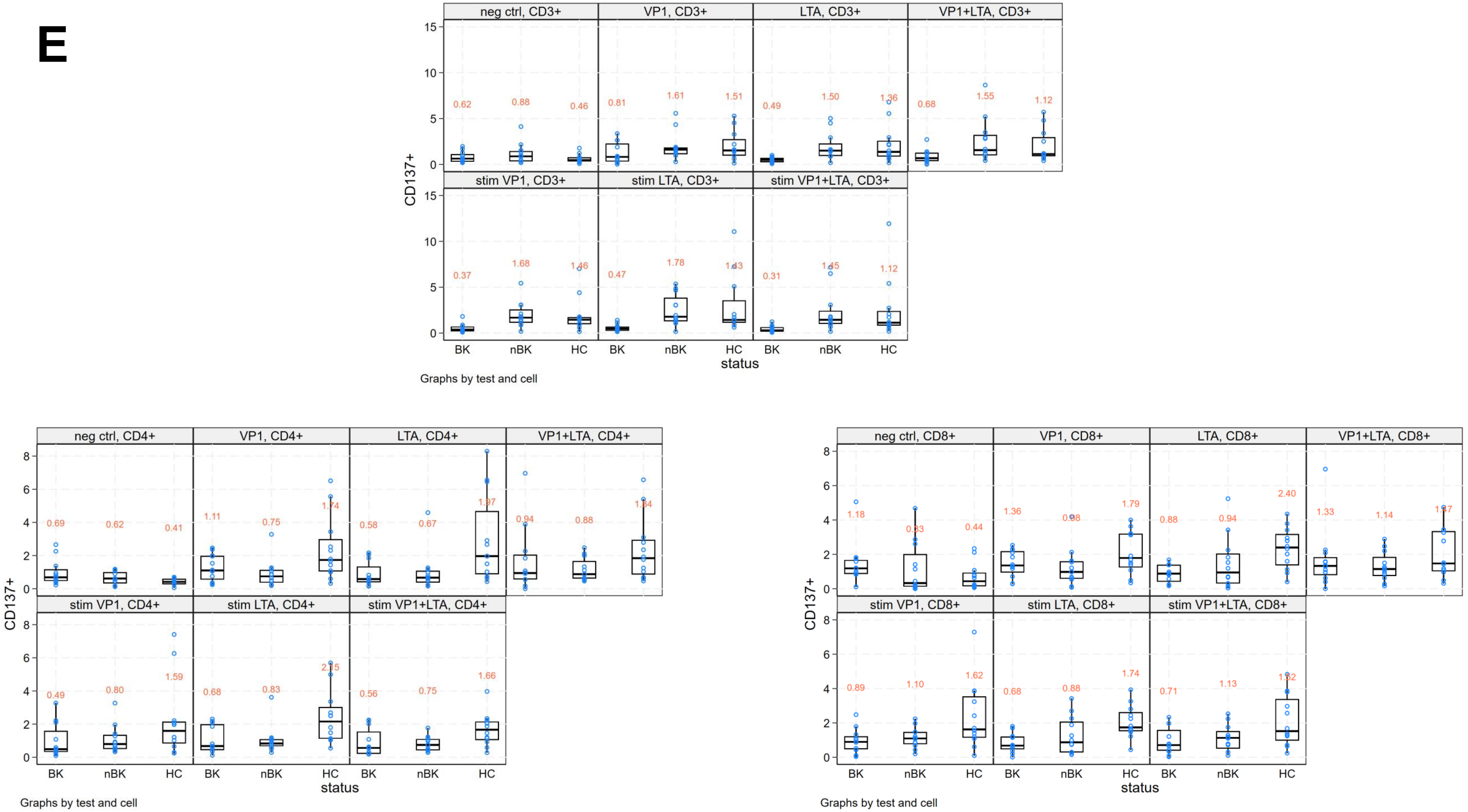
neg ctrl, CD3+
VP1, CD3+
LTA, CD3+
VP1+LTA, CD3+
stim VP1, CD3+
stim LTA, CD3+
stim VP1+LTA, CD3+
CD137+
BK nBK HC
status
Graphs by test and cell
neg ctrl, CD4+
VP1, CD4+
LTA, CD4+
VP1+LTA, CD4+
stim VP1, CD4+
stim LTA, CD4+
stim VP1+LTA, CD4+
CD137+
status
Graphs by test and cell
neg ctrl, CD8+
VP1, CD8+
LTA, CD8+
VP1+LTA, CD8+
stim VP1, CD8+
stim LTA, CD8+
stim VP1+LTA, CD8+
CD137+
status
Graphs by test and cell

F

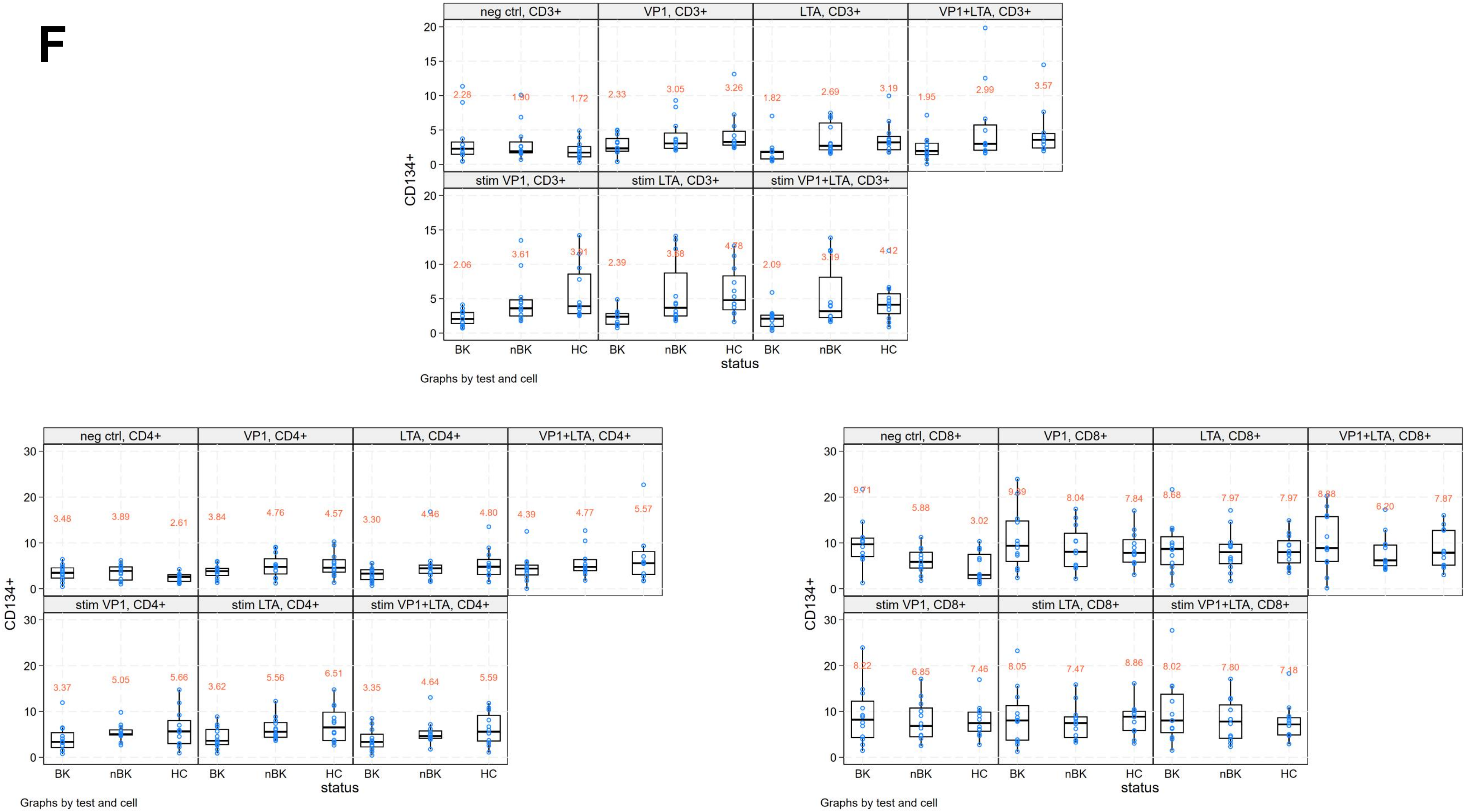
neg ctrl, CD3+
VP1, CD3+
LTA, CD3+
VP1+LTA, CD3+
stim VP1, CD3+
stim LTA, CD3+
stim VP1+LTA, CD3+
2.28
1.90
1.72
2.33
3.05
3.26
1.82
2.69
3.19
1.95
2.99
3.57
2.06
3.61
3.91
2.39
3.68
4.78
2.09
3.19
4.12
CD134+
BK
nBK
HC
status
Graphs by test and cell
neg ctrl, CD4+
VP1, CD4+
LTA, CD4+
VP1+LTA, CD4+
stim VP1, CD4+
stim LTA, CD4+
stim VP1+LTA, CD4+
3.48
3.89
2.61
3.84
4.76
4.57
3.30
4.46
4.80
4.39
4.77
5.57
3.37
5.05
5.66
3.62
5.56
6.51
3.35
4.64
5.59
neg ctrl, CD8+
VP1, CD8+
LTA, CD8+
VP1+LTA, CD8+
stim VP1, CD8+
stim LTA, CD8+
stim VP1+LTA, CD8+
9.71
5.88
3.02
9.39
8.04
7.84
8.68
7.97
7.97
8.88
6.20
7.87
8.22
6.85
7.46
8.05
7.47
8.86
8.02
7.80
7.18

G

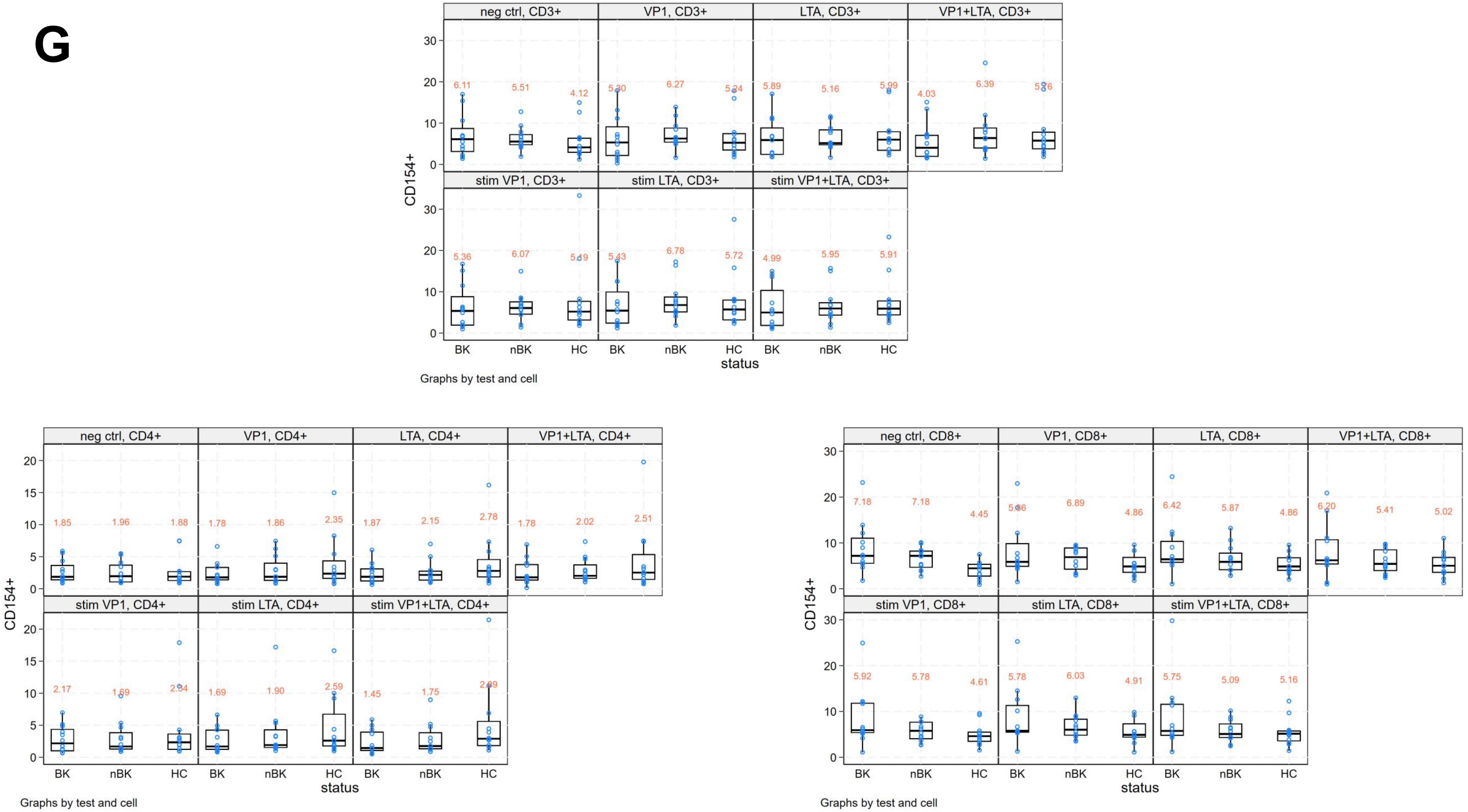
neg ctrl, CD3+
VP1, CD3+
LTA, CD3+
VP1+LTA, CD3+
stim VP1, CD3+
stim LTA, CD3+
stim VP1+LTA, CD3+
6.11
5.51
4.12
5.30
6.27
5.24
5.89
5.16
5.99
4.03
6.39
5.76
5.36
6.07
5.19
5.43
6.78
5.72
4.99
5.95
5.91
CD154+
BK
nBK
HC
status
Graphs by test and cell
neg ctrl, CD4+
VP1, CD4+
LTA, CD4+
VP1+LTA, CD4+
stim VP1, CD4+
stim LTA, CD4+
stim VP1+LTA, CD4+
1.85
1.96
1.88
1.78
1.86
2.35
1.87
2.15
2.78
1.78
2.02
2.51
2.17
1.69
2.34
1.69
1.90
2.59
1.45
1.75
2.69
CD154+
BK
nBK
HC
status
Graphs by test and cell
neg ctrl, CD8+
VP1, CD8+
LTA, CD8+
VP1+LTA, CD8+
stim VP1, CD8+
stim LTA, CD8+
stim VP1+LTA, CD8+
7.18
7.18
4.45
5.86
6.89
4.86
6.42
5.87
4.86
6.20
5.41
5.02
5.92
5.78
4.61
5.78
6.03
4.91
5.75
5.09
5.16
CD154+
BK
nBK
HC
status
Graphs by test and cell

H

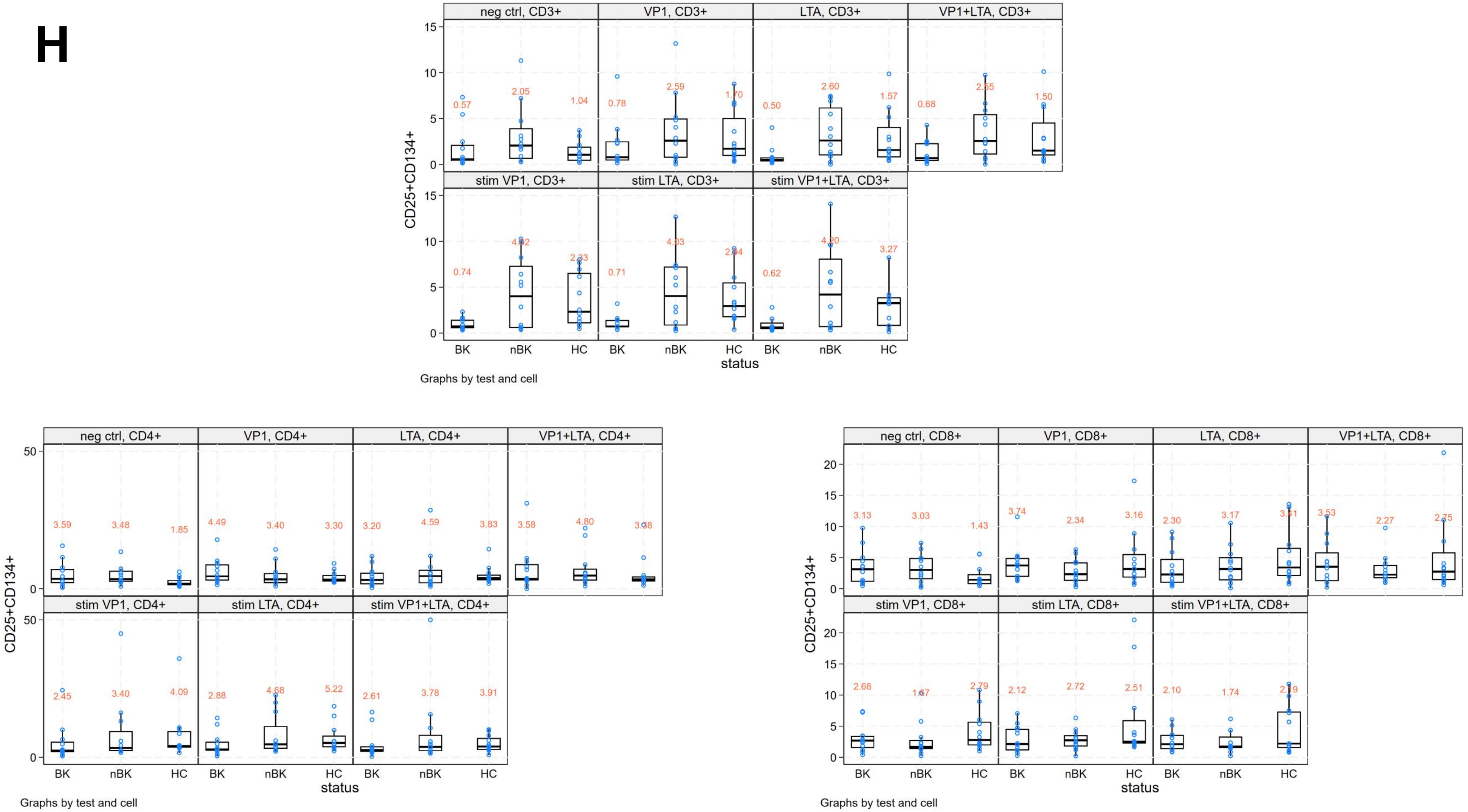
neg ctrl, CD3+
VP1, CD3+
LTA, CD3+
VP1+LTA, CD3+
stim VP1, CD3+
stim LTA, CD3+
stim VP1+LTA, CD3+
CD25+CD134+
0.57
2.05
1.04
0.78
2.59
0.50
2.60
1.57
0.68
1.50
0.74
0.71
0.62
3.27
BK
nBK
HC
status
Graphs by test and cell
neg ctrl, CD4+
VP1, CD4+
LTA, CD4+
VP1+LTA, CD4+
stim VP1, CD4+
stim LTA, CD4+
stim VP1+LTA, CD4+
CD25+CD134+
3.59
3.48
1.85
4.49
3.40
3.30
3.20
4.59
3.83
3.58
4.80
2.45
3.40
4.09
2.88
4.68
5.22
2.61
3.78
3.91
BK
nBK
HC
status
Graphs by test and cell
neg ctrl, CD8+
VP1, CD8+
LTA, CD8+
VP1+LTA, CD8+
stim VP1, CD8+
stim LTA, CD8+
stim VP1+LTA, CD8+
CD25+CD134+
3.13
3.03
1.43
3.74
2.34
3.16
2.30
3.17
3.53
2.27
2.75
2.68
2.12
2.72
2.51
2.10
1.74
BK
nBK
HC
status
Graphs by test and cell

I

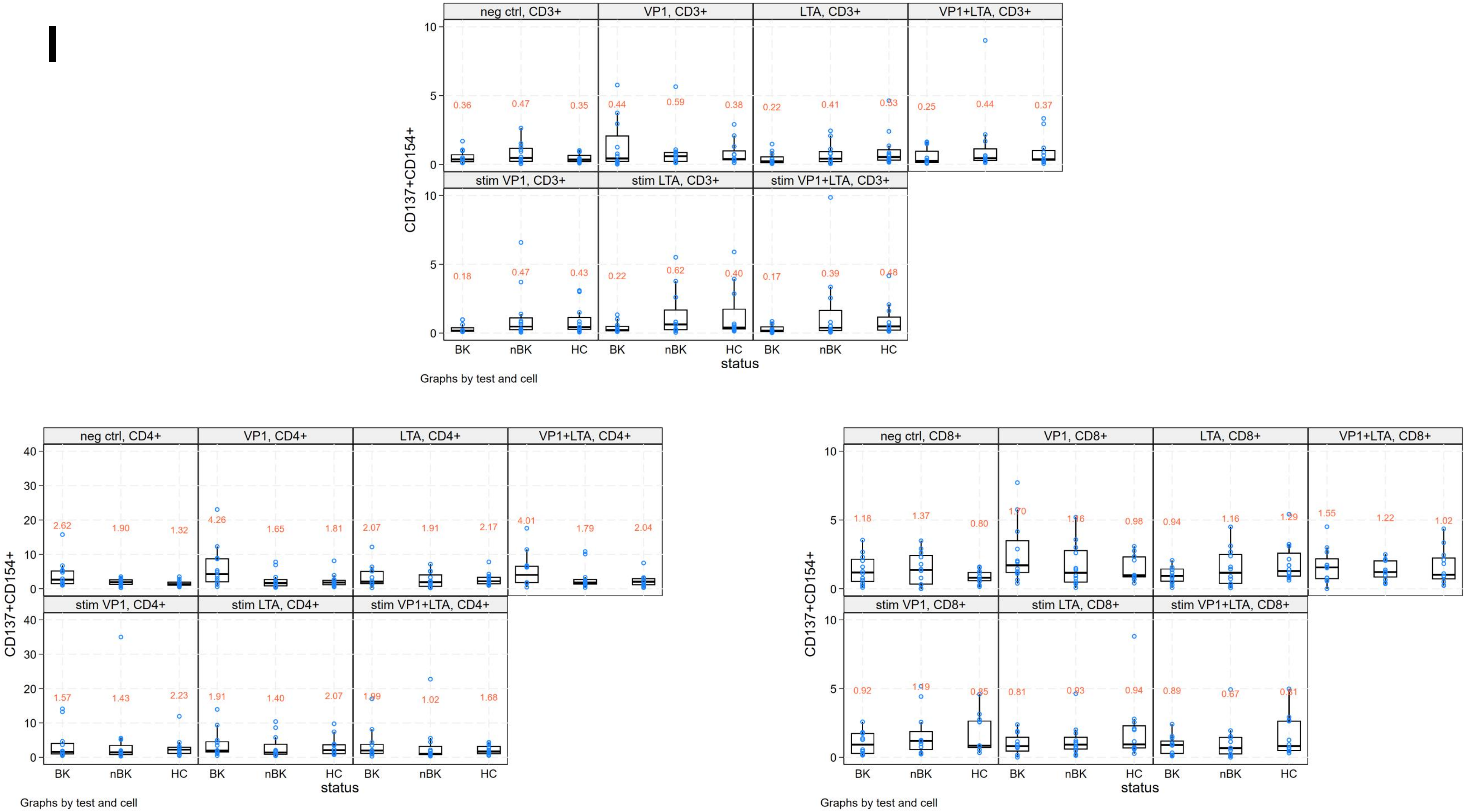
neg ctrl, CD3+
VP1, CD3+
LTA, CD3+
VP1+LTA, CD3+
stim VP1, CD3+
stim LTA, CD3+
stim VP1+LTA, CD3+
0.36 0.47 0.35
0.44 0.59 0.38
0.22 0.41 0.53
0.25 0.44 0.37
0.18 0.47 0.43
0.22 0.62 0.40
0.17 0.39 0.48
CD137+CD154+
BK nBK HC
status
Graphs by test and cell
neg ctrl, CD4+
VP1, CD4+
LTA, CD4+
VP1+LTA, CD4+
stim VP1, CD4+
stim LTA, CD4+
stim VP1+LTA, CD4+
2.62 1.90 1.32
4.26 1.65 1.81
2.07 1.91 2.17
4.01 1.79 2.04
1.57 1.43 2.23
1.91 1.40 2.07
1.99 1.02 1.68
CD137+CD154+
BK nBK HC
status
Graphs by test and cell
neg ctrl, CD8+
VP1, CD8+
LTA, CD8+
VP1+LTA, CD8+
stim VP1, CD8+
stim LTA, CD8+
stim VP1+LTA, CD8+
1.18 1.37 0.80
1.70 1.16 0.98
0.94 1.16 1.29
1.55 1.22 1.02
0.92 1.19 0.85
0.81 0.93 0.94
0.89 0.67 0.81
CD137+CD154+
BK nBK HC
status
Graphs by test and cell

Supplementary Figure S3: Heatmaps illustrating the p-values for differences in cell percentages of intracellular cytokine and surface marker expression following BKPyV antigen stimulation (Wilcoxon Rank Sum test). Patients with BKPyV viremia (BK) exhibit the lowest percentages, followed by those with non- BKPyV viremia (nBK) and healthy controls (HC). Lighter colors represent lower p-values, using stimulated-to-unstimulated ratio.

LTA; large T antigen, VP1; viral capsid protein 1 antigen, stim; co-stimulated with CD28 and CD49d antibodies

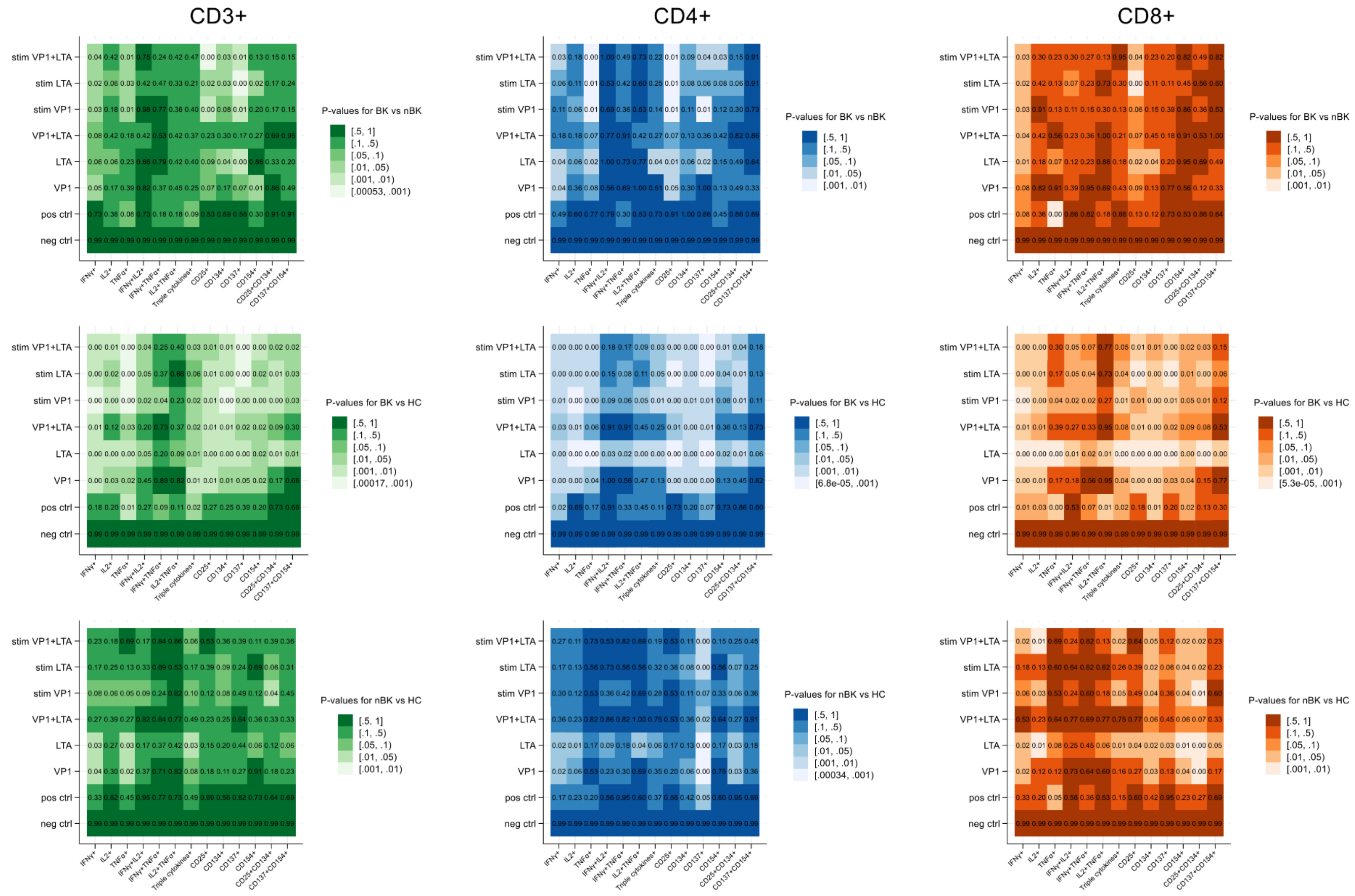

Supplement: Supplementary file 1 [file Data_Sheet_1.pdf]
